# ﻿A taxonomic review of the order Mantodea in Korea based on morphology and DNA barcodes

**DOI:** 10.3897/zookeys.1206.123355

**Published:** 2024-07-02

**Authors:** Jaeil Shim, Jeong-Hun Song

**Affiliations:** 1 Department of Agricultural Biology, National Institute of Agricultural Sciences, Wanju 55365, Republic of Korea National Institute of Agricultural Sciences Wanju Republic of Korea; 2 Department of Biology, Chungnam National University, Daejeon 34134, Republic of Korea Chungnam National University Daejeon Republic of Korea

**Keywords:** DNA barcodes, Korea, Mantodea, review, taxonomy

## Abstract

A taxonomic study of Korean Mantodea using morphological and molecular characters (COI) is presented. Eight species [*Amantisnawai* (Shiraki, 1908), *Acromantisjaponica* Westwood, 1889, *Mantisreligiosasinica* Bazyluk, 1960, *Statiliamaculata* (Thunberg, 1784), *Tenoderaangustipennis* Saussure, 1869, *T.sinensis* Saussure, 1871, *Hierodulachinensis* Werner, 1929, *H.patellifera* (Audinet-Serville, 1838)] belonging to six genera in three families are recognized. Interspecific genetic divergence of COI using uncorrected *p*-distance ranged from 6.7% to 22.4%, while intraspecific divergence ranged from 0% to 2.2% among eight Korean Mantodea species. All eight species were each strongly supported as a single lineage using COI on both neighbor-joining and parsimony trees. An illustrated key, redescriptions, habitus photographs, and illustrations of diagnostic characters of the species of Korean Mantodea are provided to facilitate identification.

## ﻿Introduction

The order Mantodea comprise approximately 2,400 species in 460 genera, making it a distinctive group of predatory insects (Ehrmann 2002; Otte et al. 2024). They exhibit remarkable diversity in morphology, hunting strategies, and habitat specialization and are widely distributed in tropical and subtropical regions (Otte et al. 2024). Members of this order are generally characterized by a carnivorous diet, mimetic behavior, triangular head, large compound eyes, raptorial foreleg with elongated forecoxa, forefemur and foretibia with characteristic spines, femoral brush for grooming, elongated pronotum, male subgenital plate (coxosternite IX), asymmetrical male genitalia, female subgenital plate (coxosternite VII), and ootheca covered by a secretion (Klass 2001; Klass and Meier 2006; Yager and Svenson 2008; Wieland 2013; Svenson et al. 2015; Hashimoto et al. 2016; Brannoch et al. 2017).

Prior to 1995, only a few entomologists had recorded four species of Mantodea on the Korean peninsula, *Mantisreligiosa* Linnaeus, 1758, *Statiliamaculata* (Thunberg, 1784), *Tenoderaangustipennis* Saussure, 1869, and *T.aridifolia* (Stoll, 1813) (Okamoto 1924; Bey-Bienko 1930; Doi 1932; Shiraki 1932; Cho 1959; Bazyluk 1960; Ju 1969; ESK and KSAE 1994). Kwon et al. (1996) recorded *Amantisnawai* (Shiraki, 1908), and Jeon et al. (1999) added three species, *Acromantisjaponica* Westwood, 1889, *Hierodulapatellifera* (Audinet-Serville, 1838), and *S.nemoralis* (Saussure, 1870), with descriptions and illustrations. Addionally, Shim et al. (2021a) reported the giant Asian mantis *Hierodulachinensis* Werner, 1929, with a diagnosis and illustrations. To date, nine species of Mantodean insects have been reported in Korea (Kim 2010, 2021; Shim et al. 2021a). While some species have been previously described, accurate identification of species within this group is challenging due to a lack of available morphological information.

In this paper, we present a taxonomic reassessment of Korean Mantodea, recognizing eight species belonging to six genera in three families, including *A.nawai* (Shiraki, 1908), *Ac. Japonica* Westwood, 1889, *M.religiosasinica* Bazyluk, 1960, *S.maculata* (Thunberg, 1784), *T.angustipennis* Saussure, 1869, *T.sinensis* Saussure, 1871, *H.chinensis* Werner, 1929, and *H.patellifera* (Audinet-Serville, 1838). Re-descriptions incorporate salient morphological features critical for accurate identification of these species, including the male genitalia. We also used molecular criteria including genetic divergence and gene tree monophyly using a COI barcode region as a multiple lines of evidence approach for the species identification. Our taxonomic review of Korean Mantodea provides redescriptions, habitus photographs, an interactive key, and diagnoses.

## ﻿Materials and methods

Studied specimens were mostly collected from inland and the islands of the Korean peninsula. Specimens were collected by direct sweeping, scanning, shifting leaf litter and light trapping. If nymphs or oothecae were found, samples were reared until the adult insect emerged. The collected specimens were killed by freezing to prevent discoloration and were moved to a drying chamber for dehydration at 60 °C for 10 days until completely hardened. The subsequent sample preparation followed methods by Brannoch et al. (2017) and Shim et al. (2021b). Briefly, the male genitalia were incubated overnight in 10% potassium hydroxide (KOH), and washed with distilled water, then 75% ethanol, before storage in glycerol. Depository of specimens examined is as follows: National Institute of Agricultural Sciences Insect Collection (**NASIC**, Wanju, Korea); Kunsan National University (KsNU, Gunsan, Korea); National Institute of Biological Resources (**NIBR**, Incheon, Korea).

The specimens were examined with a stereomicroscope (MS5, Leica Microsystem, Wetzlar, Germany). Images were obtained using a Canon DSLR (EOS 5D; Tokyo, Japan) with an attached Canon MP-E 65 mm f/2.8 1–5× lens. Several layers of photographs were combined in Helicon Focus 5.3 software (Helicon Soft Ltd, Kharkov, Ukraine) and edited using Adobe Photoshop CC 2020 (Adobe, San Jose, CA, USA). Measurements were recorded in millimeters using digital Vernier calipers (CD-15APX; Mitutoyo, Sakado, Japan). The terminology of taxonomic characters and measurements of specimens mainly followed Wieland (2013) and Brannoch et al. (2017) for external morphology, and Klass (1997) and Schwarz and Roy (2019) for male genitalia.

The following abbreviations are used for the foreleg spination formula and male genitalia: spination formula: **Avfs** = anteroventral femoral spines; **Avts** = anteroventral tibial spines; **Ds** = discoidal spines of forefemur; **Pvfs** = posteroventral femoral spines; **Pvts** = posteroventral tibial spines. Male genitalia: **afa** = anterior process of left phallomere (phalloid apophysis); **aafa** = anterior lobe of phalloid apophysis; **fda** = posterior lobe of right phallomere; **loa** = posterior membranous lobe of left phallomere; **pafa** = posterior lobe of phalloid apophysis; **L4B** = a sclerite of left phallomere, mostly spoon-shaped; **maa** = medial arm process of right phallomere; **paa** = elongated process of left phallomere, titillator; **pia** = process arising from the midlenth of the ventral wall of right phallomere, located posterolateral area of pva, strongly sclerotized; **pva** = process arising from midlenth of the ventral wall of right phallomere, located anteromesal area of pia, strongly sclerotized; **sdpl** = lateral secondary distal process; **sdpm** = median secondary distal process.

The following abbreviations are used for the provinces of Korean peninsula (Specimens examined): **GW**: Gangwon-do; **GG**: Gyeonggi-do; **CB**: Chungcheongbuk-do; **CN**: Chungcheongnam-do; **GB**: Gyeongsangbuk-do; **GN**: Gyeongsangnam-do; **JB**: Jeollabuk-do; **JN**: Jeollanam-do; **JJ**: Jeju-do (Is.).

For the study of molecular characters, we included a total of 74 specimens for DNA extraction in the dataset and the specimens used are listed in Suppl. material 1. DNA extraction, sequencing and alignments follow the methods described by Shim et al. (2021b). The mitochondrial COI was selected. Primers and amplification strategies are provided in Shim et al. (2021b). Data from GenBank for 50 foreign specimens were incorporated into the study, as indicated in Suppl. material 1. Parsimony (PA) analyses were conducted using MEGA X (Kumar et al. 2018) with 1000 bootstrap replications. A neighbor-joining analysis (NJ) was performed in MEGA X (Kumar et al. 2018) using the Kimura-2-Parameter (K2P) model (Kimura 1980). Bootstrap support values for each node were evaluated via MEGA X with 1000 replicates. Intra- and inter-specific distances in the different taxonomic levels were calculated using an uncorrected pairwise distance method (Srivathsan and Meier 2012).

## ﻿Taxonomic accounts

### ﻿Key to species of Mantodea in Korea

**Table:** 

1	(A) Pvts fully decumbent	***Acromantisjaponica***
–	(B) Pvts not fully decumbent	**2**
	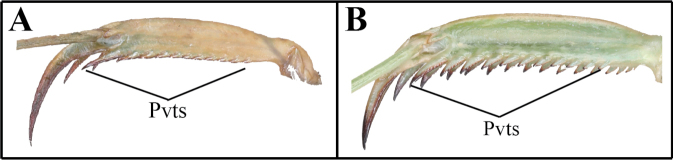	
2	(A) Genicular spurs length as long as Pvfs; second Avfs greatly enlarged	***Amantisnawai***
–	(B) Genicular spurs length clearly shorter than Pvfs; second Avfs not enlarged	**3**
	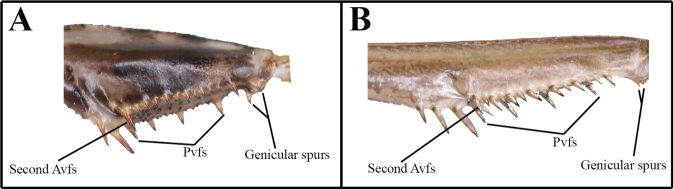	
3	(A) Forecoxa with a distinct dark or eye spot covering ~ 1/4 to 2/5 of the total length	**4**
–	(B) Forecoxa without spot or a faint dark spot covering ~ 1/9 of the total length	**5**
	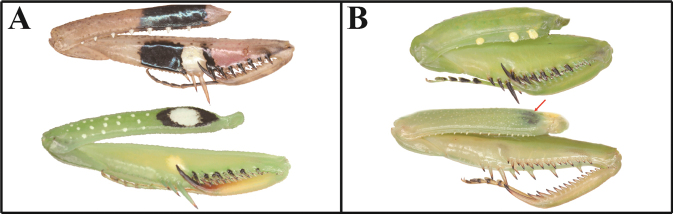	
4	(A) Tibial spur groove only with yellow spot or no spot at all	***Mantisreligiosasinica***
–	(B) Tibial spur groove with white spot, and dark patch either side	***Statiliamaculata***
	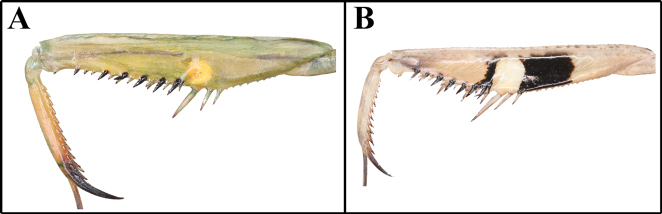	
5	(A) Pronotum long and robust; forewing without stigma pattern; abdominal sternites with longitudinally striped pattern	**6**
–	(B) Pronotum clavate in shape; forewing with stigma pattern; abdominal sternites without striped pattern	**7**
	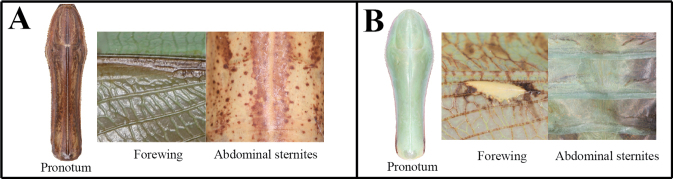	
6	(A) Forecoxa base color yellow (in life); hindwing, area of arculus near cells colored dark brown, subcostal to cubitus area brown to dark brown	***Tenoderasinensis***
–	(B) Forecoxa base color orange (in life); hindwing, area of arculus near cells transparent, subcostal to cubitus area reddish	***T.angustipennis***
	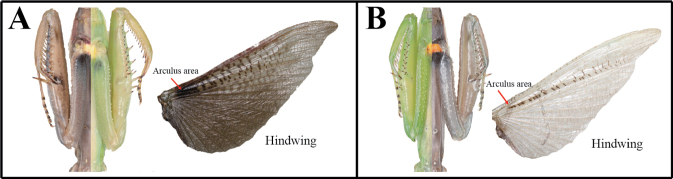	
7	(A) Furcasternite without pattern	***Hierodulachinensis***
–	(B) Furcasternite with two-band pattern	***H.patellifera***
	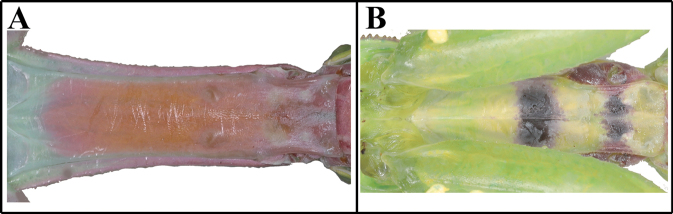	

### ﻿Species descriptions

**Family Gonypetidae Westwood, 1889**

**Subfamily Iridopteryginae Giglio-Tos, 1915**

#### ﻿Genus Amantis

Taxon classificationAnimaliaMantodeaGonypetidae

Giglio-Tos, 1915

A957ED24-1447-5E0B-9FDF-7123A82C3028

Cimantis Giglio-Tos, 1915: 154 (synonymized by Beier 1935: 28).
Shirakia Beier, 1935: 47.

##### Type species.

Mantis (Oxypilus) reticulata De Haan, 1842.

##### Diagnosis.

Very small sized mantises. Body with mottled dark spot pattern. Pronotum short, kite- shaped, its dorsal surface with longitudinal striped pattern. Genicular spurs length as long as Pvfs length. Second Avfs is as long as second Ds, much longer than the neighboring spines. Wings brachypterous or macropterous.

#### ﻿ Amantis nawai

Taxon classificationAnimaliaMantodeaGonypetidae

(Shiraki, 1908)

2BC70BAA-05BD-51C8-8E7F-215E5E8D4908

Figs 1–8 , 9–14 , 15–19

Gonypeta nawai Shiraki, 1908: 47.
Gonypeta maculata Shiraki, 1911: 318.
Amantis nawai (Shiraki, 1908): ESK and KSAE 1994: 44; Kim 2010: 31; Kim 2021: 65. Korean record.
Iridopteryx maculatus (Shiraki, 1911): Kwon et al. 1996: 221. Korean record.

##### Specimens examined.

[**NASIC] South Korea: GN**: 1♂1♀, Mt. Noja, Dongbu- myeon, Island Geojedo, Geoje-si, 7 VIII 2019, Yeong-Hun Kim; 3♂3♀, Mt. Noja, Dongbu- myeon, Island Geojedo, Geoje-si, 7 VIII 2019, Woojin Jang; **JN**: 3♂1♀, Island Yeoseodo, Yeoseo-ri, Cheongsan-myeon, Wando-gun, 26 I 2019, Jaeil shim (reared from Ootheca); **JJ**: 1♀, Andeok Valley, Seogwipo-si, Jeju-do, X 2019, Do-yoon Kim; 2♂1♀, Donnaeko, Seogwipo-si, Jeju-do, 9 IX 2020, Yeong-Hun Kim; 3♂2♀, Seonheul-ri, Jocheon-eup, Jeju-si, Jeju-do, 19 V 2023, Jaeil Shim (reared from ootheca); 2♀, Gamsan-ri, Andeok-myeon, Seogwipo-si, Jeju-do, 23 IX 2023, Jaeil Shim; 1♀, Gamsan-ri, Andeok-myeon, Seogwipo-si, Jeju-do, X 2023, Jisung Kim; **Japan**: 1♂, Yanbaru, Okinawa, 1–4 I 2020, Wonjun Seong, Forest.

##### Redescription.

**Figures 1–8.**
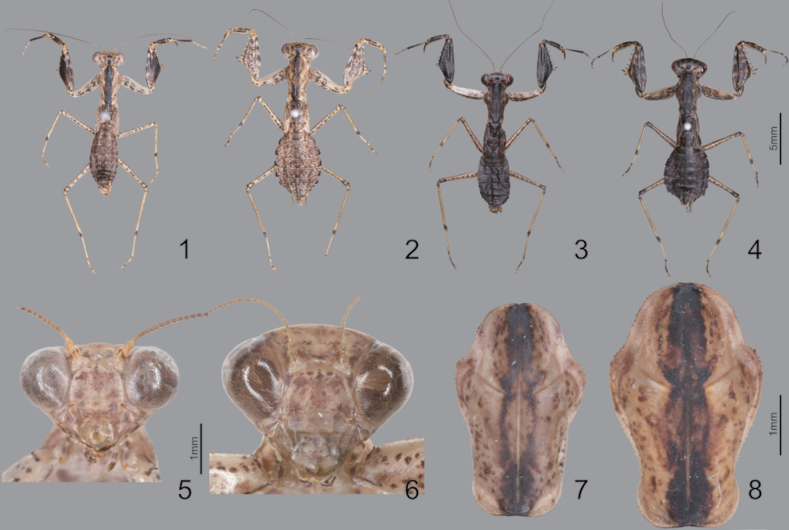
Habitus, head and pronotum of *Amantisnawai***1** male dorsal aspect (Geojedo island) **2** female dorsal aspect (Geojedo island) **3** male dorsal aspect (Jeju) **4** female dorsal aspect (Jeju) **5** male face **6** female face **7** male pronotum **8** female pronotum.

**Figures 9–14.**
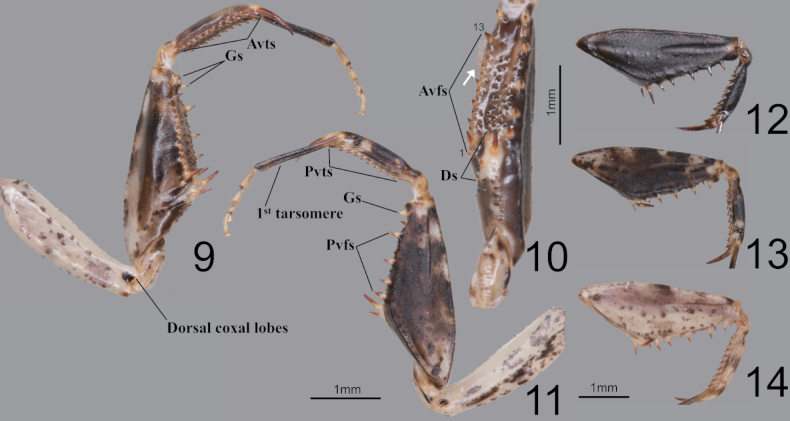
Male foreleg of *Amantisnawai***9** ventral aspect **10** interior aspect **11** dorsal aspect **12** foreleg dorsal aspect (Jeju) **13** foreleg dorsal aspect (Geojedo island) **14** foreleg dorsal aspect (Yeoseodo island). White arrow = femoral brush.

**Figures 15–19.**
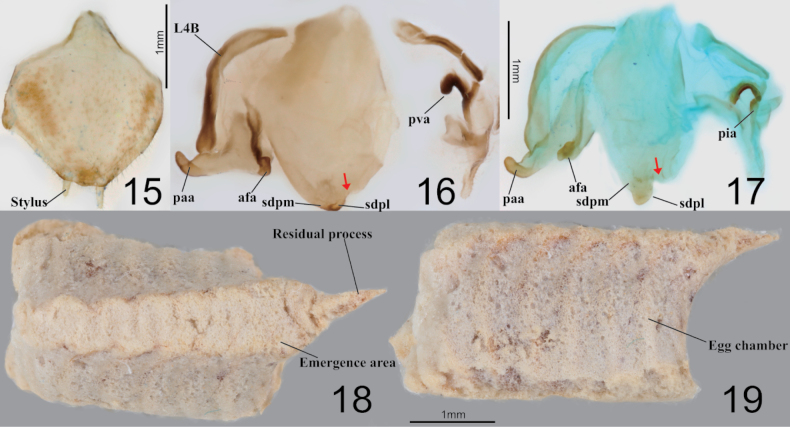
Male genitalia and ootheca of *Amantisnawai***15** subgenital plate **16** male genitalia (Jeju) **17** male genitalia (Yeoseodo island) **18** ootheca (dorsal aspect) **19** ootheca (lateral aspect). Red arrows = concave area.

***Measurements* (mm)**: Body length ♂ 12.1–13.0, ♀ 13.8–15.2; head width ♂ 2.2, ♀ 3.3; head length ♂ 2.9, ♀ 3.9; pronotum width ♂ 2.1, ♀ 2.5; pronotum length ♂ 3.3, ♀ 3.9; forewing (tegmina) length ♂♀ 1.6. **Male** (Figs 1, 3, 5, 7, 9–17). Very small sized mantises. ***Coloration*** (Figs 1, 3): Body color bright brown to dark brown. Body surface with irregular dark brown spots. ***Head*** (Fig. 5): Triangular, slightly broader than pronotum maximum width. Head length 1.3× width. Antenna filiform. Antenna nearly 3× as long as pronotum. Vertex nearly flat, posterior area (Figs 1, 3) with five dark spots. Compound eye slightly protruding, its surface glossy and brown moire pattern (in live specimens). Ocelli small, all of the same shape and size. Epistomal sulcus weakly concave. ***Prothorax*** (Fig. 7): Dorsal surface with dark brown longitudinal stripe pattern. Pronotum very short, kite-shaped, lateral margin with minute denticulation. Pronotum length 1.4× as long as maximum width. Metazone nearly 2× as long as prozone. ***Forelegs* (*Prothoracic legs*)** (Figs 9–17): Coxa and femur slightly longer than pronotum. Coxa dorsal margin (Figs 9, 11) with 9–13 minute forecoxal spines. Dorsal and ventral coxal lobes (Fig. 9) fully divided from each other; dorsal coxal lobes with dark brown spot. Femur, tibia, and first tarsomere darker than body color (Figs 1, 3, 11). Femur interior surface (Fig. 10) with numerous denticles. Genicular spurs (Figs 9, 11) well-developed, as long as Pvts length. Spination formula (Figs 9–11): Avts = 10–12; Pvts = 12–13; Avfs = 11–14; Pvfs = 4; Ds = 4. In 13 Avfs (Figs 9, 10): spines 2 and 13 larger than remaining Avfs; spines 10, 11, and 12 smallest of Avfs. ***Meso- and metathoracic legs***: Meso- and metathoracic legs long and slender, apical area of tibia and first tarsomere dark brown. Tarsi 5-segmented. First tarsomere of mesotarsus subequal to combined length of remaining segments, first tarsomere of metatarsus longer than remaining segments combined. ***Wings***: Brachypterous. Wing venation faded. ***Abdomen***: Cerci setose, not flattened, with nine or ten segments. Male subgenital plate (coxosternite IX) (Fig. 15) nearly rhombus, inter-stylar margin slightly convex; stylus rather short. ***Male genitalia*** (Figs 16, 17): Right phallomere with C-shaped pva (Fig. 16); pia sclerotized and weakly wrinkled; fda distal edge margin rounded, surface with few setae. Left phallomere with short and curved paa; afa sclerotized, rectangular (slightly concave at the middle), shape of distal end variable, surface weakly granulated; Korean populations with two types of afa: dorsally curved type (Fig. 16) and posteriorly decumbent type (Fig. 17); loa mostly invisible. Ventral phallomere nearly oblong; sdpm very weakly developed; sdpl slightly expanded (digitiform), right margin of sdpl conspicuously concave. **Female** (Figs 2, 4, 6, 8). Similar to male, with following differences. ***Head*** (Fig. 6): length 1.2× width. Antenna nearly 2× as long as pronotum. Vertex swollen. ***Prothorax*** (Fig. 8): Pronotum length 1.5× maximum width. Narrowest width on posterior one-third of pronotum. ***Forelegs* (*Prothoracic legs*)** (Figs 2, 4): Femur color generally same as body color. First tarsomere distal end area dark brown. ***Abdomen***: Post-lateral margin of tergite with dark brown spot. **Ootheca** (Figs 18, 19). ***Measurements* (mm)**: Length 3.2–5.3; width 2.8–3.4; height 2.7–3.4; length of emergence area 3.9–6.4; width of emergence area 1.0–1.6. ***Identification***: Prism-shaped, triangular in cross-section. Ootheca attached by ventral surface. External wall colored russet. External coating (Figs 18, 19) beige, consisting of a very thin layer and frothy material, lateral surface and emergence area fully covered. Exhibiting ~ 4–8 egg chambers (Fig. 19). Lateral surface with longitudinal parallel ridges (boundaries). Distal end of ootheca truncate and rough. Distal flap area with long residual process (Fig. 18). **Nymph. *First instar nymph***: Body surface shiny, reddish brown with brightly colored transverse pattern on body segments and legs; postero-medial edge of pro-, meso-, and metathorax tergite slightly protruding.

##### Biological notes.

*Amantisnawai* is distributed throughout the southern islands of the Korean peninsula, as reported by Kwon et al. (1996). This species is commonly found beneath the litter layer in shaded areas and typically lays its oothecae under dead leaves or between stones on the ground. The species exhibits unique behaviors, including the vibration of its antenna and swinging of its forelegs. The first instar nymphs usually hatch between mid-June and early July, while adults emerge in mid-August.

##### Distribution.

China, Japan, Taiwan, South Korea.

##### Remarks.

*Amantisnawai* occurs in East Asia (Yamasaki 1981; Chou 2006; Patel and Singh 2016; Oshima 2018). Korean populations of *A.nawai* exhibit two distinct male genitalia structures, distinguished by the ‘afa’ structure (Figs 16, 17), as well as variable forefemur color patterns (Figs 12–14). The Yeoseodo Island population features a posteriorly decumbent ‘afa’ structure (Fig. 17), and the male forefemur color matches their body color (Fig. 14). In contrast, the Geojedo and Jeju Island populations exhibit a dorsally curved ‘afa’ structure (Fig. 16), and the forefemur color of most male samples is darker than their body color (Figs 1, 3). However, there is no significant intraspecific genetic divergence (COI) between the Yeoseodo and Geojedo Island populations. Additionally, the Jeju population shows only a 0.3% divergence from the other islands.

**Family Hymenopodidae Giglio-Tos, 1915**

**Subfamily Acromantinae Brunner de Wattenwyl, 1893**

#### ﻿Genus Acromantis

Taxon classificationAnimaliaMantodeaHymenopodidae

Saussure, 1870

8AF0DABF-1286-596B-B086-47AF9828EC66

##### Type species.

*Mantisoligoneura* De Haan, 1842

##### Diagnosis.

Small-sized mantises. Compound eye with stripe pattern (in live specimens). Anterior area of vertex and lower frons slightly protruding. Pronotum metazone middle area with flat bulge. Genicular spurs clearly shorter than the shortest Pvfs length. Pvts fully decumbent. Meso- and metathoracic femora with weakly expanded postero-ventral femoral lobe. Posterior margins of sternites with a medial lip. Hindwing apex distally truncate.

#### ﻿ Acromantis japonica

Taxon classificationAnimaliaMantodeaHymenopodidae

Westwood, 1889

EB175968-BDCA-5ACD-B8EA-D7D04F3D0C30

Figs 20–29 , 30–33

Acromantis japonica Westwood, 1889: 43.
Acromantis japonica Westwood, 1889: Jeon et al. 1999: 226 (South Korea); Kim 2021: 65. Korean records.

##### Specimens examined.

[**NASIC] South Korea: GN**: 2♀, Mt. Noja, Dongbu-myeon, Island Geojedo, Geoje-si, 10 IX 2016, Jaeil Shim; 1♂, Mt. Wangjo, Nambu-myeon, Tappo-ri, Island Geojedo, Geoje-si, 23 XII 2019, Jun-Ho Lee; 2♀, Mt. Noja, Dongbu-myeon, Island Geojedo, Geoje-si, 29 IX 2016, Byeongmin Jeong; **JN**: 3♂, Jeongdo-ri, Wando-gun, 10 I 2019, JaeDong Gim (reared from ootheca); 3♂, Jeongdo-ri, Wando-gun, 26 II 2019, Jaeil Shim (reared from ootheca); 8♂6♀, Is. Bogil-do, Bogil-myoen, Wando-gun, 2 III 2023, Jaeil Shim (reared from ootheca); **JJ**: 1♂, Mt. Sambang, Namjeju-gun, Jeju-si 28 IX 2000, Miae Kim; 1♂, Hwasun-ri, Andeok-myeon, Seogwipo-si, 12–13 IX 2023, Jaeil Shim; 8♂1♀, Sumang-ri, Namwon-eup, Seogwipo-si, 13–14 IX 2023, Jaeil Shim.

##### Redescription.

**Figures 20–29.**
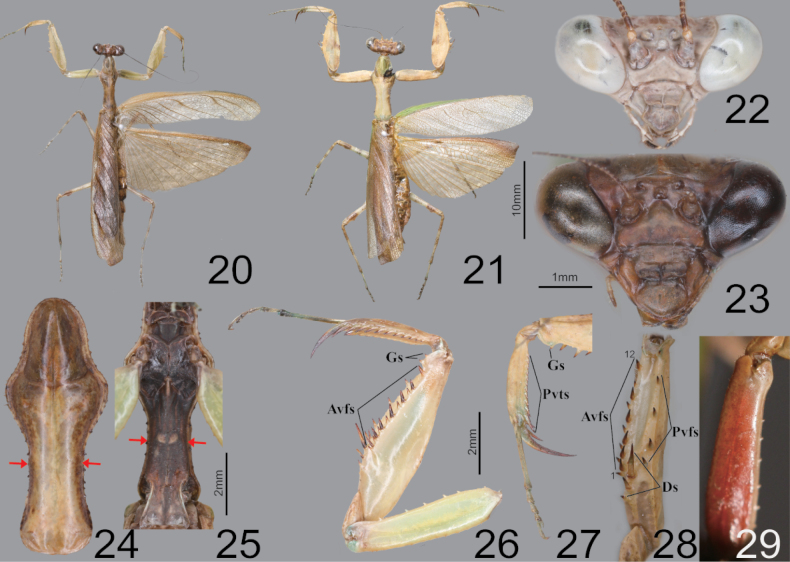
Habitus, head, pronotum and foreleg of *Acromantisjaponica***20** male dorsal aspect **21** female dorsal aspect **22** male face **23** female face **24** female pronotum **25** male furcasternite **26** male foreleg ventral aspect **27** foreleg tibia and tarsus (dorsal aspect) **28** foreleg interior aspect **29** female coxa ventral aspect (live specimens). Red arrows = bulge and protuberance.

**Figures 30–33.**
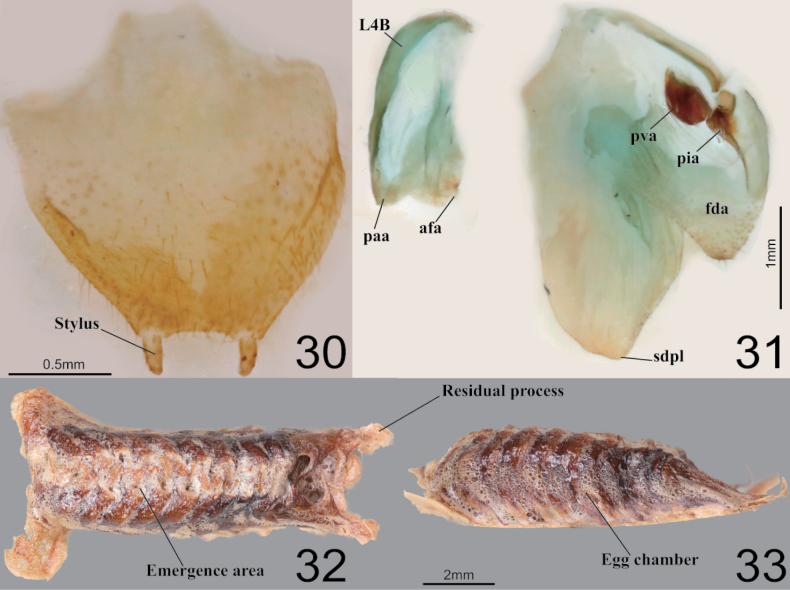
Male genitalia and ootheca of *Acromantisjaponica***30** subgenital plate **31** male genitalia **32** ootheca (dorsal aspect) **33** ootheca (lateral asepct).

***Measurements* (mm)**: Total length (vertex to tip of abdomen) ♂ 23.3–26.2, ♀ 28.4–32.2; head width ♂ 4.1–4.2, ♀ 5.0–5.1; head length ♂ 2.4, ♀ 3.4; pronotum width ♂ 2.6–2.7, ♀ 3.4; pronotum length ♂ 6.5–7.0, ♀ 8.2–8.4; forewing (tegmina) length ♂ 17.4–20.0, ♀ 17.0–18.5. **Male** (Figs 20, 22, 24–28, 30, 31). Small sized mantises. ***Coloration*** (Fig. 20): Body greenish brown to brown. ***Head*** (Fig. 22): Triangular; width 1.7× length. Antenna > 2× as long as pronotum. Vertex nearly flat, anterior vertex with weakly pronounced spurs. Postocellar tubercle weakly pronounced. Compound eye globular, slightly protruding laterally; compound eye with brightly coloured moire pattern (in live specimens). Ocelli larger than female, middle ocellus nearly globular, lateral two ocelli oblong. Posterior apex of lower frons (Fig. 22) with protruding spur. ***Prothorax*** (Figs 24, 25): Pronotum length 2.5–2.6× maximum width. Pronotum (Fig. 24) surface smooth, lateral margin with few denticles; metazone > 2× longer than prozone; middle of metazone with flat bulge (pair); metazone lateral margin slightly concave. Furcasternite (Fig. 25) very slightly convex, middle area with flat protuberance. ***Forelegs* (*Prothoracic legs*)**: Foreleg (Figs 20, 26) surface smooth and shiny, pale green. Coxa dorsal margin with 6–10 spines. Dorsal and ventral coxal lobes (Fig. 26) fully divided from each other. Femur dorsal margin (Figs 20, 26) slightly convex at the middle, distal half of margin very slightly concave. Genicular spurs (Fig. 26) well developed, conspicuously shorter than Pvfs, postero-ventral genicular spurs weakly curved outward. Pvts (Fig. 27) fully decumbent. Spination formula: Avts = 12–13; Pvts = 12; Avfs = 10–13; Pvfs = 4; Ds = 4. In 12 Avfs (Fig. 26, 28): spines 2, 4, 6, 8, 10, and 12 size larger than remaining Avfs, dark brown. Ds 2 and 3 dark brown in live specimens. ***Meso- and metathoracic legs***: Meso- and metathoracic legs with transverse dark pattern. Meso- and metathoracic femora (Fig. 20) with weakly expanded postero-ventral femoral lobe. Tarsi 5-segmented. ***Wing***: Wings completely surpassing the end of abdomen (Fig. 20). Forewing costal area bright brown or green, discoidal area brownish and transparent, cubitus brown. Forewing with oblique brown stripes. Hindwing costal area brown, discoidal area brownish and transparent; hindwing apex brown, truncate. ***Abdomen***: Sternites III–VII posterior margin with a medial lip, process slightly expanded. Cerci setose, 13 segments. Male subgenital plate (coxosternite IX) (Fig. 30) irregularly rounded rhomboidal in shape, inter-stylar margin generally concave, but medial area occasionally slightly convex. Ventral surface of subgenital plate with numerous setae. Styli rather short. ***Male genitalia*** (Fig. 31): Right phallomere pia sclerotized and surface with few denticles; fda nearly triangular in shape, its surface with few setae. Left phallomere, paa nearly absent (very blunt); afa weakly sclerotized, and distal end bulbous; loa invisible. Ventral phallomere nearly elliptical, without any melanized structures, distal margin truncate or weakly bilobed (sdpm and sdpl may be expanded). **Female** (Figs 21, 23, 29). Similar to male, with following differences. ***Head*** (Fig. 23): width 1.5× length. Antenna as long as pronotum. ***Prothorax***: Pronotum length 2.4× maximum width. ***Forelegs* (*Prothoracic legs*)**: Foreleg dorsal surface brown, coxa ventral surface (Fig. 29) red in live specimens. ***Wings*** (Fig. 21): Forewing costal area color green. **Ootheca** (Figs 32, 33). ***Measurements* (mm)**: Length 10.7–14.8; maximum width 4.3–4.8; maximum height 3.6–4.3; length of emergence area 7.8–10.4; width of emergence area 1.5–2.4. ***Identification***: Nearly rectangular dorsally, hemispherical in cross-section, each edge with slightly expanded attachment area (Fig. 32). Ootheca attached on ventral surface. External wall color brown (Fig. 33). External coating bright brown but surface weakly covered. Exhibiting ~ 10–15 egg chambers (Fig. 33) clearly delimited by prominently visible curved lips. Distal end softly truncate and weakly rough. **Nymph. *First instar nymph***: Body color black and surface shiny (ant mimic); compound eye with white stripes (moiré pattern); posterior margin of pro-, meso-, and metathoracic tergite with white lines; tarsus of meso- and metathoracic legs white. ***Second to last instar nymph***: Body brown to dark brown with brightly colored mottled pattern; vertex spurs well developed; abdomen with expanded lamellar process (lip) in the middle of sternites.

##### Biological notes.

*Acromantisjaponica* is found in the southern islands of the Korean Peninsula (Jeon et al. 1999; An 2011). They occur in leaf litter, under broad leaves and on tree trunks. Adults exhibit positive phototaxis, and oothecae are typically laid under stones and the bark of rotten logs. This species overwinters in the egg stage inside their oothecae (Taniguchi 1987; Befu 1992). They exhibit behaviors such as vibrating their antennae and swinging forelegs. Nymphs fold their abdomens back during rest. The first instar nymphs hatch from the end of May to early June, while adults emerge beginning from the end of August.

##### Distribution.

China, Japan, Taiwan, North Korea, South Korea.

##### Remarks.

The genus *Acromantis* has characteristic stripe patterns on their compound eyes when they are alive (see Nakamine et al. 2017: figs 2, 3), weakly pronounced postocellar tubercles and truncated hindwings (Vyjayandi et al. 2010; Mukherjee et al. 2017; Shcherbakov and Anisyutkin 2018). They occur in East Asia to Australasia (Jeon et al. 1999; Nakamine 2016; Patel et al. 2016).

**Family Mantidae Latreille, 1802**

**Subfamily Mantinae Latreille, 1802**

#### ﻿Genus Mantis

Taxon classificationAnimaliaMantodeaMantidae

Linnaeus, 1758

0BC5EFFB-FB26-5E58-8172-C04B389965D0

Mantes Geoffroy, 1762: 399.

##### Type species.

Gryllus (Mantis) religiosa Linnaeus, 1758

##### Diagnosis.

Medium to large sized mantises. Body color mostly bright green, yellow, brown. Male antenna reddish, conspicuously thicker than the female’s. Vertex swollen. Forecoxal proximal area with dark spot, ventral surface with numerous yellow spots. Tibial spur groove area with yellow spot. Furcasternite with numerous spots. Metathoracical episternum with a dark lateral spot. Male inter-stylar margin notched.

#### ﻿ Mantis religiosa sinica

Taxon classificationAnimaliaMantodeaMantidae

Bazyluk, 1960

08B16809-A834-5AE6-BE33-F9F51A48D048

Figs 34–46 , 47–51

Mantis religiosa sinica Bazyluk, 1960: 255.
Mantis religiosa sinica Bazyluk, 1960: 255; Kim 2010: 31; Kim 2021: 65. Korean record.
Mantis religiosa (Linnaeus, 1758): ESK and KSAE 1994: 44. Korean record.

##### Specimens examined.

[**NASIC] South Korea: GW**: 3♂, Hotel Ramada, Daegwallyeong Pass, Pyeongchang-gun, 22 VIII 2019, JuHyeong Sohn; **GG**: 1♀, Jeongok-eup, Yeoncheon-gun, 22 VIII 84, Sunhee Jang; 1♀, Mt. Acha, Gwangjin-gu, Seoul, 8 IX 1977, Sunhee Yoon, Sungshin Univ.; 1 Nymph 1♂4♀, Island Gureopdo, Gureop-ri, Deokjeok-myeon, Incheon, 17 VII 2019, Byeongmin Jeong (reared from nymph); 2♂1♀, Island Gureopdo, Gureop-ri, Deokjeok-myeon, Incheon, 28 VI 2023, Jaeil Shim, Wonjun Sung (reared from nymph); **CN**: 1♂1♀, Coastal Dune, Sindu-ri, Wonbuk-myeon, Taean-gun, 1 IX 2001, Haechul Park, near the grassland; 4♂6♀, Sonhwang-ri, Woongcheon-eup, Boryeong-si, 12 VII 2023, Jaeil Shim, near the grassland (reared from nymph); 2♂3♀, Coastal Dune, Sindu-ri, Wonbuk-myeon, Taean-gun, 12 VIII 2023, Jaeil Shim, near the grassland; **GB**: 2♂, Mt. Angi, Songhyeon-dong, Andong-si, VIII 2022, Jaeil Shim; 3 Nymphs, Gyeongjeong Beach, Gyeongjeong-ri, Chuksan-myeon, Yeongdeok-gun, 20 VII 2023, Jaeil Shim; **GN**: 2♀, Mt. Noja, Dongbu- myeon, Island Geojedo, Geoje-si, 15 IX 2021, Jaeil Shim; **JB**: 1♂, Byeonsan-myeon, Buan-gun, VIII 1999, Jeonbuk Nat. Univ; 1♂, Mt. Jeoksang, Muju-gun, 8 IX 1999, Taewoo Kim; 1♀, Kunsan Nat. Univ., Gunsan-si, 31 VIII 2019, JuHyeong Sohn; 6♂8♀, Is. Yamido, Okdo-myeon, Gunsan-si, 5 VII 2023, Jaeil Shim (reared from nymph); **JJ**: 3 Nymphs, Gwangchigi Beach, Goseong-ri, Seongsan-eup, Seogwipo-si, 16 V 2021, Jaeil Shim; **Hungary**: *Mantisreligiosareligiosa*, 1♂, Mt. Csakyar, Vertes, 23 VIII 2003, J.C. Sohn, Haraszt hegy 250m.

##### Redescription.

**Figures 34–46.**
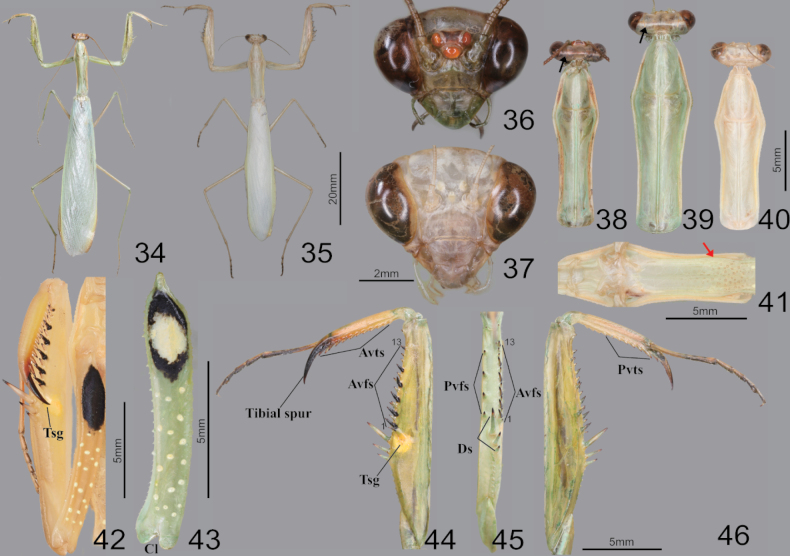
Habitus, head, pronotum and foreleg of *Mantisreligiosasinica***34** male dorsal aspect **35** female dorsal aspect **36** male face **37** female face **38** male pronotum **39** female pronotum (inland) **40** female pronotum (Gureopdo island) **41** furcasternite **42** foreleg ventral aspect (live specimens) **43** coxa ventral aspect **44** forleg ventral aspect **45** foreleg interior aspect **46** foreleg dorsal aspect. Abbreviation: Tsg = tibial spur groove. Red arrows = dark spots of furcasternite.

**Figures 47–51.**
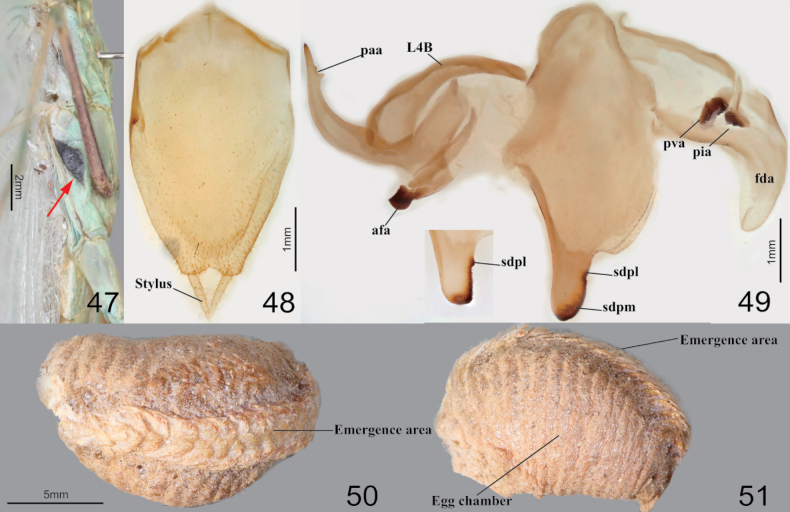
Metathorax, male genitalia and ootheca of *Mantisreligiosasinica***47** metathorax episterum **48** subgenital plate **49** male genitalia (small box = variation of sdpl) **50** ootheca (dorsal aspect) **51** ootheca (lateral asepct). Red arrow = dark pattern.

***Measurements* (mm)**: Total length (vertex to tip of abdomen) ♂ 42.3–55.2, ♀ 50.8–72.4; head width ♂ 5.1–5.4, ♀ 6.2–6.5; head length ♂ 3.8–4.2, ♀ 5.4–5.6; pronotum width ♂ 4.0–4.2, ♀ 5.6–6.0; pronotum length ♂ 13.2–14.0, ♀ 16.6–18.0; forewing (tegmina) length ♂ 38.4–43.2, ♀ 36.1–47.7. **Male** (Figs 34, 36, 38, 42–49) Medium to large sized mantises. ***Coloration*** (Figs 34, 35): Body bright green, brown and yellow. ***Head*** (Fig. 36): width 1.3× length. Vertex swollen; with pale transverse magenta line along dorsal apex (in live specimens) (Fig. 38). Compound eye globular, anteriorly protruding; dorsal surface with two transverse stripes (in live specimens). Ocelli large, oval, pale yellow. Antenna filiform; pedicel, scape and initial flagellum pale, remaining flagellum orangish to reddish brown (Figs 34, 36). Antenna length > 2× as long as pronotum, conspicuously thicker than in female. Lower frons posterior apex weakly protruding; pale transverse line at posterior one-third of lower frons (in live specimens). Epistomal sulcus transverse. ***Prothorax*** (Fig. 38): Pronotum flattened dorso-ventrally, its length 3.3× as long as maximum width. Prozone lateral margin with small denticles. Metazone 3× as long as prozone; lateral margin smooth. Medial keel protruding. Furcasternite (Fig. 41) posterior area with numerous dark spots. ***Forelegs* (*Prothoracic legs*)** (Figs 42–46): Coxa dorsal margin with 6–11 spines (Fig. 43), small denticles located between its spines. Coxa ventral surface (Figs 42, 43) with large oblong black spot or eye spot in proximal area; remaining surface with 15–24 yellow spots, center of each spot with small seta. Coxal lobes fully divided from each other. Tibial spur groove (Figs 42, 44) with yellow spotted (blotch) pattern. Genicular spurs minute. Ventral surface of tibia (Fig. 44) yellow to orange. Spination formula (Figs 44, 45): Avts = 12–13; Pvts = 7; Avfs = 12–13; Pvfs = 4; Ds = 4. In 13 Avfs (Figs 44, 45): spines 2, 4, 6, 8, 10, 12 and 13 larger and black, spines with black spot at the base. Tarsus with ventral area brownish. ***Meso- and metathorax and legs***: Metathorax episternum (Fig. 47) with dark triangular spot. Meso- and metathoracic legs long and slender; tarsi 5-segmented. ***Wings***: Forewing completely surpassing the end of abdomen. Stigma elongate, slightly protruding; color same as forewing venation. Forewing anterior margin brown, discoidal area mostly hyaline. Hindwing transparent but apex brownish. ***Abdomen***: Cerci setose, elongated, and thick, not flattened, brown; 17 segments. Male subgenital plate (coxosternite IX) (Fig. 48) irregularly elliptical in shape, inter-stylar margin notched in V-shape. Styli rather long. ***Male genitalia*** (Fig. 49): Right phallomere smooth, forming a C-shaped pva; pia sclerotized and weakly wrinkled; fda triangular. Left phallomere with elongate and curved paa, its distal end sharp and sclerotized, paa anterior margin one-fourth to one-third with one or two short projections; afa strongly sclerotized, rounded, anterior margin obliquely curved dorsally, surface granulated; L4B curved spoon-shaped. Ventral phallomere (Fig. 49) nearly rhomboidal; sdpm elongated, blunt finger-like and slightly curved dorsally, distal end and right margin sclerotized and surface with numerous denticulation; sdpl slightly protruding (blunt projection). **Female** (Figs 35, 37, 39, 40). Similar to male, with following differences. ***Head*** (Fig. 37): Vertex convex. Head width 1.1× as long as length. Antenna as long as pronotum. ***Prothorax*** (Figs 39, 40): Pronotum length 2.9–3.0× as long as maximum width. Metazone 2–3× as long as prozone. **Ootheca** (Figs 50, 51). ***Measurements* (mm)**: Length 17.2–25.1; maximum width 11.4–14.3; maximum height 9.3–10.5; length of emergence area 17.0–21.6; width of emergence area 3.2–5.3. ***Identification***: Oblong, nearly hemispherical in cross-section. External wall bright brown to brown (Fig. 50). External coating beige on egg chamber surface, pale on emergence area (flap). Exhibiting ~ 20–30 egg chambers (Fig. 51) clearly delimited by visible slightly curved lips. Distal end of ootheca narrowed into residual process; residual process attached to substrate. **Nymph. *First instar nymph***: Body brown, vertex dorsal apex with pair of dark spots. ***Mid to last instar nymph***: In brown morph, dorsal surface of body with a few stripes.

##### Biological notes.

*Mantisreligiosasinica* is sparsely distributed on the Korean Peninsula. This species prefers broad grasslands and bushy areas, comprised of shrubs and grass in sandy fields as its habitat. It exhibits positive phototaxis, meaning it is attracted to light. When threatened, it makes a hissing sound by rubbing its hindwings against the abdomen. The first instar nymphs hatch from the end of May onwards while the adults emerge in August.

##### Distribution.

China, Japan, South Korea.

##### Remarks.

*Mantisreligiosa* (Linnaeus) is a widely distributed Paleotropical and Holarctic species (Lia 2007; Patel and Singh 2016; Otte et al. 2024). Bazyluk (1960) and Roy (1968) provided illustrations of the sdpm variations and Schwarz and Roy (2019) presented figures of male genitalia. Additional morphological information was provided by Ehrmann and Borer (2015), Shcherbakov and Anisyutkin (2018), and Shcherbakov and Govorov (2020). Bazyluk (1960) classified this species as the subspecies *M.religiosasinica*, which is found in East Asia including Korea. The population on Gureopdo Island exhibits a shorter pronotum, smaller body size, and shorter forewing length compared to inland populations. However, there are minimal genetic differences in the partial COI regions (0%–0.2%). Furthermore, all specimens were supported as a single lineage using COI on both NJ and PA trees (Fig. 138).

#### ﻿Genus Statilia

Taxon classificationAnimaliaMantodeaMantidae

Stål, 1877

4009479D-5A90-5E3B-80C5-8103525AA2CA

##### Type species.

*Pseudomantisnemoralis* Saussure, 1870

##### Diagnosis.

Medium sized mantises. Vertex nearly flat. Pronotum slender. Ventral surface of foreleg shiny, densely patterned, forcoxa proximal area with dark pattern. Tibial spur groove with whitish yellow to yellow spots. Male inter-stylar margin notched. Male genitalia: sdpl and sdpm well developed, angled at 90°.

#### ﻿ Statilia maculata

Taxon classificationAnimaliaMantodeaMantidae

(Thunberg, 1784)

18662287-A970-5836-BAB0-A077B4713DC3

Figs 52–62 , 63–70

Mantis maculata Thunberg, 1784: 61.
Pseudomantis haanii Saussure, 1871a: 37, 1871b: 400.
Statilia maculata var. hyalina Giglio-Tos, 1927: 410.
Statilia haani var. major Werner, 1922: 154.
Statilia maculata continentalis Werner, 1935: 495.
Statilia maculata (Thunberg, 1784): ESK and KSAE 1994: 44; Kim 2010: 31; Kim 2021: 65. Korean record.
Statilia nemoralis (Saussure, 1870): Jeon et al. 1999: 227 (misidentification); Kim 2010: 31 (misidentification). Korean record.

##### Specimens examined.

[**KsNU] South Korea: CN**: 1♂, Mt. Bongsoo, Daechung-myeon, Yesan-gun, 18 V 2017 Hongjoon Choi; **JB**: 1♀, Eunpa Lake, Jigok-dong, Gunsan-si, 3 IX 2016; Donghwan Na; 1♀, Miryong-dong, Gunsan-si, 27 VI 2017, Eunhye Jeon; [**NASIC] South Korea: GW**: 1♂, Ssangyong, Yeongwol-gun, 30 IX 1999, Miae Kim; **GG**: 1♀, Mt. Umyeon, Seocho-gu, Seoul, 1 IX 1991, Jiyung Oh; 1♀, Jamsil 6-dong, Songpa-gu, Seoul, 20 X 1997, Soyeon Kim; 1♀, Mt. Nam, Hada-ri, Heungcheon-myeon, Yeoju-si, 3 IX 2000, Yeongbo Lee; 1♀, Seodun-dong, Suwon-si, 4 IX 2000, Taewoo Kim; 1♂, Seodun-dong, Suwon-si, 20 IX 2000, Taewoo Kim; 1♂, Seodun-dong, Suwon-si, 20 IX 2001, Jeonghun Hwang; ♂1, Haguidong, Uiwang-si, 24 IX 2003, Jeongsun Lee; 1♂, Mt. Gwanggyo, Jangan-gu, Suwon-si, 10 X 2003, Mikyeong Ahn; **CB**: 1♀, Mt. Nam, Cheongju-si, 8 VIII 2000, Hyea Lee; 1♂, Geumseok-ri, Geumwang-eup, Eumseong-gun, 14 IX 2019, Seong-Gyu Lee; **CN**: 1♂1♀, Coastal Dune, Sindu-ri, Wonbuk-myeon, Taean-gun, 1 IX 2005, Yeongbo Lee; **GB**: 1♀, Mt. Sobaek, Punggi-gun, 5 IX 2000, Miae Kim; 1♂3♀, Mt. Angi, Songhyeon-dong, Andong-si, VIII 2022, Jaeil Shim; 2♂6♀, Hotel Interburgo, Manchon-dong, Suseong-gu, Daegu-si 29 X 2023, Jaeil Shim; **GN**: 1♂, Sinhyeon-ri, Geoje island, Geoje-si, 11 IX 2002, Miae Kim; 2♂1♀, Mundong Waterfall, Geoje-si, 19 IX 2006, Miae Kim; ♀1 (green), Recreational Forest, Dongbu-myeon, Geojedo, Geoje-si, 29 IX 2020, Wonjun Sung; **JB**: 2♂4♀, Gueok, Yongjin-myeon, Wanju, 13 VIII 2013, Hanjun Bae; 1♂, Iseo-myeon, Wanju-gun, 26 VII 2016, Taeman Han; 1♀, Iseo-myeon, Wanju-gun, 29 VII 2014, Kyusuk Lee; 1♂1♀, Apartment, Iseo-myeon, Wanju-gun, 6 VIII 2015, Kyusuk Lee; 1♀ (green), National Institute of Agricultural Sciences, Iseo-myeon, Wanju-gun, 24 VIII 2016, Hyeop Lee; 1♀, Miryong-dong, Gunsan-si, 27 VI 2017, Eunhye Jeon; 2♂, Iseo-myeon, Wanju-gun, 23 VIII 2019, Jaeil Shim; 1♂2♀, Mt. Moak, Gui-myeon, Wanju-gun, 7 IX 2019, Jaeil Shim; 7♂5♀, Jangsu-eup, Jangsu-gun, 5 IX 2019, Jaeil Shim; **JN**: 3♀, Geumgok-dong, Buk-gu, Gwangju-si, 20 III 2021, Jaeil Shim; 4♀, Near Yeosu Airport, Sinpung-ri, Yulchon-myeon, Yeosu-si, IX 2021, Byeongmin Jeong; 2♂1♀, Is. Bogil-do, Bogil-myoen, Wando-gun, 2 III 2023, Jaeil Shim (reared from ootheca); 8♂9♀, Island Yeoseodo, Yeoseo-ri, Cheongsan-myeon, Wando-gun, VI 2023, Jaeil shim (reared from Ootheca); **JJ**: 1♂, Seonheul-ri, Jeju-si, 28 IX 2000, Taewoo Kim; 1♂, Sinae 1-ri, Jeju-si, 18 X 2001, Mikyeong Ahn; 1♂, Bijarim Forest, Jeju-si, 22 IX 2006, Miae Kim; **Japan**: 1♂, Asakura, Kyushu, 24 VIII 2013, Sangwook Park; [**NIBR] South Korea: GG**: 1♀, Namhansanseong, Seongnam, 28 IX 1997, Jeong Yun Chang.

**Figures 52–62.**
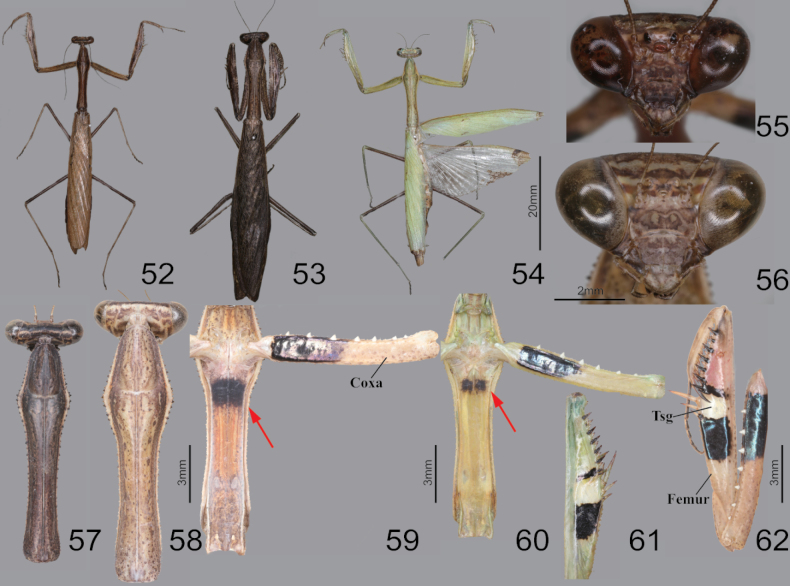
Habitus, head, pronotum and foreleg of *Statiliamaculata***52** male dorsal aspect **53** female dorsal aspect **54** female dorsal aspect (green morpho-type) **55** male face **56** female face **57** male pronotum **58** female pronotum **59** furcasternite (brown morpho-type) **60** furcasternite (green morpho-type) **61** femur ventral aspect (green morpho-type) **62** foreleg ventral aspect (live specimens). Abbreviation: Tsg = tibial spur groove. Red arrows = dark spot of furcasternite.

##### Redescription.

**Figures 63–70.**
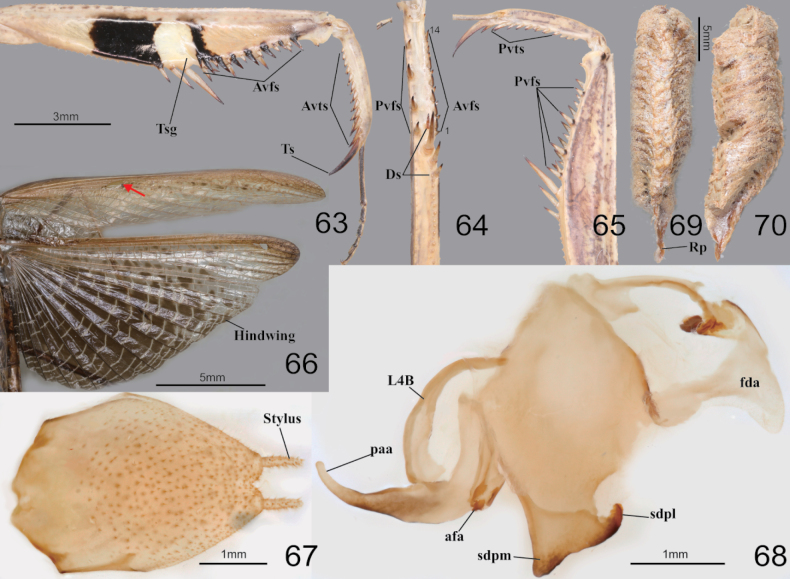
Foreleg, wings, male genitalia and ootheca of *Statiliamaculata***63** foreleg ventral aspect **64** foreleg interior aspect **65** foreleg dorsal aspect **66** wings **67** subgenital plate **68** male genitalia **69** ootheca (dorsal aspect) **70** ootheca (lateral asepct). Abbreviation: Ts = tibial spur Tsg = tibial spur groove. Red arrow = stigma.

***Measurements* (mm)**: Total length (vertex to tip of abdomen) ♂ 35.2–43.1, ♀ 43.7–57.7; head width ♂ 4.5–5.0, ♀ 5.4–5.8; head length ♂ 2.9–3.2, ♀ 4.1–4.3; pronotum width ♂ 2.9–3.2, ♀ 4.4–4.6; pronotum length ♂ 12.1–14.0, ♀ 14.0–15.3; forewing (tegmina) length ♂ 26.4–31.2, ♀ 33.1–36.1. **Male** (Figs 52, 55, 57, 63–68) Medium sized, body slender. ***Coloration*** (Figs 52, 53): Beige to dark brown. ***Head*** (Fig. 55): Triangular. Head width 1.5–1.6× length. Vertex flat; dark transverse stripe on dorsal apex. Vertex and juxtaocular bulge with sparse, pale, transversely striped pattern. Compound eye large, globular, protruding laterally; in live specimens, dorsal and lateral surface with two brightly colored lines. Ocelli large, oblong. Antenna filiform, slightly longer than pronotum. Lower frons posterior apex protruding very slightly. Epistomal sulcus slightly concave. ***Prothorax*** (Fig. 57): Pronotum slender, narrow; length 4.1–4.3× as long as maximum width; dorsal surface smooth. Pronotum lateral margin with numerous denticles. Medial keel protruding. Metazone 3× as long as prozone. Membranous area between basis of forecoxa attachment, shiny and pinkish pearl in color (in live specimens). Furcasternite (Fig. 59) anterior area with rectangular dark spot, remaining posterior area reddish brown to magenta. ***Forelegs* (*Prothoracic legs*)** (Figs 62–65): Coxa very slender, triangular; dorsal margin (Figs 59, 62) with 6–8 white spines; proximal area of ventral surface (Fig. 59) with large and shiny rectangular black spot. Coxal lobes divided from each other. Femur ventral surface shiny, with two black spots (Figs 62, 63), large rectangular spot preceding the tibial spur groove, linear spot (transverse line) distad to tibial spur grooves; tibial spur groove (Fig. 63) with white or whitish yellow pattern; distal area of ventral surface (Fig. 62) pale pink to magenta (in live specimens). Spination formula: Avts = 11; Pvts = 6–7; Avfs = 14; Pvfs = 4; Ds = 4. Tarsomere ventral surface (euplantula) dark brown. Basal rim of Pvts with dark spot. In 14 Avfs (Figs 63, 64): spines 2, 4, 6, 8, 10, 12, and 14 larger in size than remaining Avfs; spines 1, 2, 4, 6, 8, 10, 12, and 14 black, with small dark spot at the base. ***Meso- and metathoracic legs***: Tarsi 5-segmented. ***Wings*** (Fig. 66): Forewing completely surpassing the end of abdomen. Stigma elongate, slightly protruding. Hindwing costal area reddish brown; discoidal area with darkish smoky mottled pattern but cross veins clearly transparent. ***Abdomen***: Cerci setose, not flattened, brown, with 14 segments. Male subgenital plate (coxosternite IX) (Fig. 67) irregular rhomboidal, inter-stylar margin deeply notched in V-shape. Styli rather long. ***Male genitalia*** (Fig. 68): Right phallomere forming a V-shape pva; pia sclerotized and weakly wrinkled; fda triangular. Left phallomere with elongate and curved paa, surface smooth, distal end blunt and slightly swollen; afa sclerotized, small and irregularly rough, surface weakly granulated; L4B C-shaped. Ventral phallomere (Fig. 68) irregular rhomboidal; sdpm sclerotized, wide triangular; sdpl anteriorly curved hook-shaped with blunt apex, margin between sdpm and sdpl flat or slightly concave. **Female** (Figs 53, 56, 58, 59). Similar to male, with following differences. ***Coloration*** (Fig. 53): Body and forewing color beige to dark brown. ***Head*** (Fig. 56): width 1.3× as long as length. Antenna 2× longer than prozone length. ***Prothorax*** (Figs 58): Pronotum length 3.1–3.3× as long as maximum width. **Green morphotype female** (Figs 54, 60, 61). ***Prothorax***: Rectangular black spot (Fig. 60) of furcasternite absent or very weakly developed, remaining posterior area greenish. ***Forlegs* (*Prothoracic legs*)** (Figs 60, 61): Tibial spur grooves (Fig. 61) with rectangular pale spotted pattern. Distal area of femur ventral surface pale pink or green. ***Wings*** (Fig. 54): Hindwing transparent or anal area with few mottled dark spots. **Ootheca** (Figs 69, 70). ***Measurements* (mm)**: Length 20.4–28.9; maximum width 6.1–10.2; maximum height 6.4–8.3; length of emergence area 15.3–24.2; width of emergence area 2.2–3.5. ***Identification***: Fusiform in shape, nearly hemispherical in cross-section. Proximal end with medial elevation of emergence area. Ootheca attached by its ventral surface. External wall bright brown. External coating weakly covering lateral zone of emergence area; beige in color. Exhibiting ~ 25–50 egg chambers clearly delimited by visible prominently oblique lips (Fig. 70). Distal end of ootheca narrowed into residual process, greatly elongated, and attached to substrate. **Nymph. *First instar nymph***: Body dark brown, leg with few brightly colored stripes. ***Mid to last instar nymph***: Forefemur distal area (pale pink to magenta) covered by mottled black or brown spots.

##### Biological notes.

*Statiliamaculata* is found throughout the Korean Peninsula. This species has shown a remarkable adaptability to urban and suburban environments, and is often observed on building walls and streetlights, exhibiting positive phototaxis. They are known to deposit their oothecae under stones and in cracks of tree bark. Notably, *S.maculata* is capable of producing a hissing sound by rubbing its hindwings and abdomen together. This species typically hatches in early June, with adults emerging in the middle of August.

##### Distribution.

China, Japan, Nepal, South Korea, Taiwan. Invasive in Eastern USA and Russia.

##### Remarks.

*Statiliamaculata* is predominantly found in the eastern Palearctic regions (*S.maculata* has also been introduced to Eastern USA and Russia), whereas *S.nemoralis* is described from the Philippines and, erroneously, from various Southeast Asian countries (Ehrmann 2002; Patel and Singh 2016; Schwarz et al. 2018). These two species, with *S.nobilis* (Brunner de Wattenwyl, 1893), are frequently confused in the taxonomic literature, leading to numerous misidentifications (see details in Schwarz et al. 2018); for example, both *S.nobilis* and *S.maculata* have been erroneously reported as *S.nemoralis* (Wang 1993; Jeon et al. 1999; Oshima 2017; Schwarz et al. 2018; Oshima 2020; Shcherbakov and Govorov 2020). Notably, the green morph is relatively uncommon in female *S.maculata* (Fig. 54) but prevalent in both sexes of *S.nobilis* and *S.nemoralis* (Zhu et al. 2012; Ehrmann and Borer 2015; Schwarz et al. 2018).

Jeon et al. (1999) initially reported *S.nemoralis* in Korea based on two female specimens lacking black spots on the furcasternite. Differences in the male genitalia, specifically the margins of the sdpl and sdpm, are reliable for distinguishing between *S.maculata* and *S.nobilis* (Schwarz et al. 2018). On the other hand, the lack of a black spot on the furcasternite is a common feature of the green morph of *S.maculata*. The occurrence of the Philippine *Statilianemoralis* is continental SE Asia needs confirmation. Subsequent examinations revealed that the specimens initially identified by Jeon et al. (1999) as *S.nemoralis* were, in fact, misidentifications of *S.maculata*.

**Subfamily Tenoderinae Brunner de Wattenwyl, 1893**

#### ﻿Genus Tenodera

Taxon classificationAnimaliaMantodeaMantidae

Burmeister, 1838

DD327FBF-7556-5FC0-8442-D5EA067A5040

Mantis (Tenodera) Burmeister, 1838: 534.
Paratenodera Rehn, 1903: 705.

##### Type species.

*Mantisfasciata* Manuel, 1797

##### Diagnosis.

Large sized mantises. Male body slender, female robust. Ventral surface of forefemur patterned with minute spots. Area between forecoxae attachment point of yellow to orange in color. Hindwing with dark mottled pattern. Abdominal sternites with yellowish longitudinal stripes at middle. Male genitalia: aafa and pafa well developed, pafa spoon- or blade-shaped; loa elongate (Jensen et al. 2010).

#### ﻿ Tenodera angustipennis

Taxon classificationAnimaliaMantodeaMantidae

Saussure, 1869

80E33C36-1749-55B4-AEE9-6DD47AF22911

Figs 71–81 , 82–87

Tenodera angustipennis Saussure, 1869: 69.
Tenodera angustipennis Saussure, 1869: ESK and KSAE 1994: 44; Kim 2010: 31; Kim 2021: 65. Korean record.

##### Specimens examined.

[**KsNU] South Korea**: **JB**: 1♀, River Geumgang, Gunsan- si, 15 IX 2012, Hyojoong Kim, near the river mouth; 1♂, Kunsan Nat. Univ., Gunsan-si, 15 X 2015, Soyeon Kim; 1♀, Eunpa Lake, Gunsan-si, 10 V 2016, Sihyun Kim; 1♀, Kunsan Nat. Univ., Gunsan-si, 20 VIII 2017, Juyeong Oh; [**NASIC] South Korea: GW**: 1♂, Songjuk Coastal area, Ganseong-eup, 19 IX 2001, Miae Kim; **GG**: 1♂, Gaepo-dong, Seoul, 1 IX 1991, Hyeonjeong Jo; 2 Nymphs, Seodun-dong, Suwon-si, 13 VIII 1998, Graduate School of Korea Univ.; 1♂, Gosaek-dong, Suwon-si, 20 VIII 1998, Seongsun Jang, ridge of rice field; 1♂, Yulgeuk 2-ri, Heungcheon-myeon, Yeoju-si, 24 VIII 1998, Yeongbo Lee, ridge of rice field; 1♂, Seodun-dong, Suwon-si, 11 IX 1998, Graduate School of Korea Univ.; 1♂1♀, Yulgeuk 2-ri, Heungcheon- myeon, Yeoju-si, 30 IX 1999, Yeongbo Lee, ridge of rice field; 1 Nmyph, Seodun-dong, Suwon-si, 6 VII 2000, Taewoo Kim; 3 Nmyphs, Seodun-dong, Suwon-si, 20 VII 2000, Taewoo Kim; 2♂1♀, Seodun-dong, Suwon-si, 20 IX 2000, Taewoo Kim; 5 Nymphs, Shihwa, Siheung-si, 30 VII 2003, Jaecheon Son; 1♂, Sinheung College, Howon-dong, Uijeongbu-si, 26 IX 2005, Jindong Yeo; 5 Nymphs, World Cup Park, Seongsan-dong, Mapo-gu, Seoul, 20 VII 2013, Yeongbo Lee; **CB**: 1♀, Mt. Nam, Cheongju-si, 24 VIII 2000, Heeah Lee; 1♀, Maebong peak, Mt. Songni, Boeun-gun, 17 IX 2002, Yeongbo Lee; 1♀, Maebong peak, Mt. Songni, Boeun-gun, 18 IX 2002, Haechul Park; 1♀, Box office, Mt. Songni, Jangam-ri, Boeun-gun, 30 IX 2002, Jaecheon Son; 1♂1♀, Gamgok-myeon, Eumseong-gun, 31 VIII 2019, Byeongmin Jeong; 1♀, Parking area of Cheongju Airport, Ipsang-ri, Naesu-eup, Cheongwon-gu, Cheongju-si, 15 IX 2023, Jaeil Shim; **CN**: 1♂, Sindu-ri, Wonbuk-myeon, Taean-gun, 1 IX 2001, Haechul Park, grassland; 1 Nymph, Sindu-ri, Wonbuk-myeon, Taean-gun, 1 IX 2005, Jaecheon Son; 2♀, Coastal Dune, Sindu-ri, Wonbuk-myeon, Taean-gun, 12 VIII 2023, Jaeil Shim, near the grassland; **GB**: 1♂, Rest area of expressway, Chilgok-gun, 8 IX 2000, Taewoo Kim; 2♀, Mt. Palgong, Daegu, 1 IX 2014, Taeman Han; 2♂1♀, Gomo station, Suseong-gu, Daegu, 3 IX 2016, Jaeil Shim; 1♂, Gomo station, Suseong-gu, Daegu, 14 IX 2019, Jaeil Shim; 1♂6♀, Mangudang park, Hyomok-dong, Daegu-si 29 X 2023, Jaeil Shim; **GN**: 2♀, Yonggang-ri, Hwagae-myeon, Hadong-gun, 15 IX 2023, Jaeil Shim; **JB**: 1♀, Jeonbuk Nat. Univ., Jeonju-si, 17 X 2017, Sunyeong Park; 1♀, Jinan-Maisan Rest area of expressway, Jinan-gun, 22 VIII 2019, Jaeil Shim; 1♂, National Institute of Agricultural Sciences, Iseo-myeon, Wanju-gun, 31 VIII 2019, Jaeil Shim; 1♀, Jangsu-eup, Jangsu-gun, 5 IX 2019, Jaeil Shim; 2♂5♀, Mt. Moak, Gui-myeon, Wanju-gun, 7 IX 2019, Jaeil Shim; 1♂, Temple Geumsansa, Gimje-gun, Gimje-si, 5 IX 2020, Jaeil Shim; **JN**: 2♀, Myeongsasimni, Coastal Dune, Island Bigeumdo, Sinan-gun, 22 IX 2001, Haechul Park; **JJ**: 1♀, Island Udo, Jeju-si, 10 X 1999, Taewoo Kim.

**Figures 71–81.**
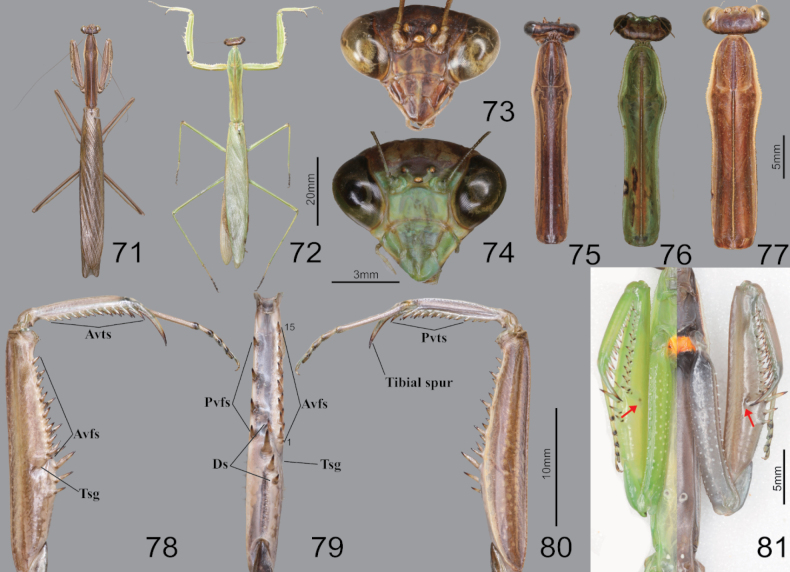
Habitus, head, pronotum and foreleg of *Tenoderaangustipennis***71** male dorsal aspect **72** female dorsal aspect **73** male face **74** female face **75** male pronotum **76** female pronotum (small sized) **77** female pronotum (large sized) **78** foreleg ventral aspect **79** foreleg interior aspect **80** foreleg dorsal aspect **81** ventral aspect of foreleg and furcasternite (live specimens). Abbreviation: Tsg = tibial spur groove. Red arrows = dark spot near the tibial spur groove.

##### Redescription.

**Figures 82–87.**
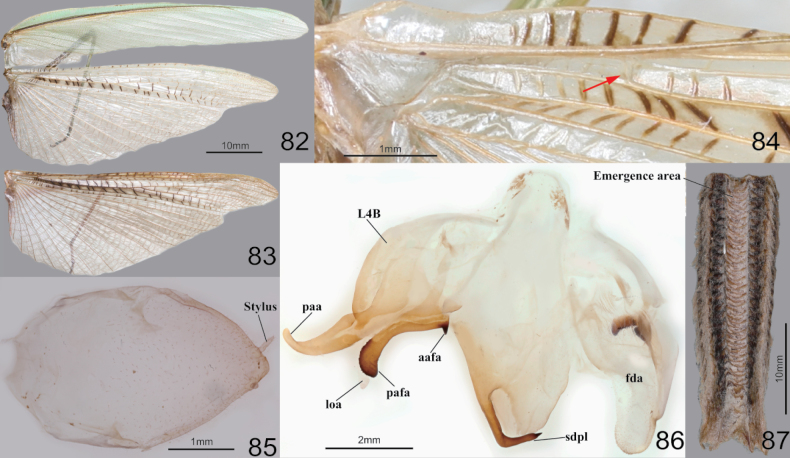
Wings, male genitalia and ootheca of *Tenoderaangustipennis*. **82** wings (green morpho-type) **83** hindwing (brown morpho-type) **84** hindwing venations **85** subgenital plate **86** male genitalia **87** ootheca (dorsal aspect). Red arrow = arculus area.

***Measurements* (mm)**: Total length (vertex to tip of abdomen) ♂ 44.2–63.3, ♀ 51.2–86.6; head width ♂ 5.9–6.5, ♀ 6.9–7.8; head length ♂ 4.5–4.9, ♀ 5.6–6.7; pronotum width ♂ 4.3–4.9, ♀ 4.9–6.4; pronotum length ♂ 18.0–22.3, ♀ 19.2–25.0; forewing (tegmina) length ♂ 38.4–48.2, ♀ 38.1–55.2. **Male** (Figs 71, 73, 75, 78–80, 82–86). Large sized, body slender. ***Coloration*** (Figs 71, 82): Body and forewing discoidal area green, greenish brown and brown. ***Head*** (Fig. 73): Triangular. Head width 1.3× as long as head length. Vertex slightly convex, brown; apex with a bright brown transverse line. Compound eye globular. Ocelli large, oblong. Antenna filiform; antenna length nearly 1.5× as long as pronotum. Lower frons posterior apex slightly protruding. Epistomal sulcus slightly concave. Lower frons with three, clypeus and labrum with two dark longitudinal stripes. ***Prothorax*** (Fig. 75): Pronotum long and slender, flatted dorso-ventrally, dorsal surface smooth; length 4.1–4.4× as long as maximum width. Prozone lateral margin with few denticulations in large sized specimens. Metazone color orangish in green morph; lateral margin smooth; metazone 3.4–4.7× as long as prozone. Between basis of forecoxa attachment membranous surface (Fig. 81) orange (in live specimen). Postcervical plate and anterior area of furcasternite with gradational dark pattern in brown morph (Fig. 81) and occasionally in green morph. Medial keel brownish (Fig. 76). ***Forelegs* (*Prothoracic legs*)** (Figs 78–81): Coxa (Fig. 81) dorsal margin with pale color, 7–10 whitish spines, conical or blunt; ventral surface with pale and dark pattern on proximal area in brown morph; remaining surface with numerous white spots. Tibial spur groove (Figs 78, 81) with faint dark spot. Spination formula (Figs 78–80): Avts = 12–17; Pvts = 9; Avfs = 14–16; Pvfs = 4; Ds = 4. In 15 Avfs (Figs 78, 79): spines 2, 4, 6, 8, 10, 12, and 15 larger than remaining Avfs; spines 1, 2, 4, 6, 8, 10, 12, and 14 with dark brown spot at the base. Each tarsomere (Fig. 78) distal area dark brown. Tarsi 5-segmented. ***Wings*** (Figs 82–84): Forewing completely surpassing end of abdomen; costal area green, discoidal area transparent. Forewing (Fig. 82) subcosta and radius brown, its color obviously darker than other veins. Hindwing anal area hyaline and nearly transparent in green morph (Fig. 82), brownish and smoky spotted pattern in brown morph (Fig. 83); arculus veins (Fig. 84) and nearby cells mostly transparent; cross veins of subcostal and cubitus area brown to dark brown. ***Abdomen***: A longitudinal yellow stripe in middle of abdominal sternites; abdominal sternites with brightly colored mottled pattern. Cerci setose, not flattened, with 17 segments. Male subgenital plate (coxosternite IX) (Fig. 85) irregular rhomboidal, inter-stylar margin extremely convex; ventral surface with numerous setae. Styli rather short. *Male genitalia* (Fig. 86): Right phallomere forming nearly V-shaped pva; pia sclerotized and weakly wrinkled; posterior surface of pia with weakly expanded membranous area, surface with minute denticulation; fda elongate lobed shape. Left phallomere (Fig. 86) with elongate and curved paa, its distal apex rounded; aafa sclerotized, straight spike shape; pafa sclerotized, curved at more than 45° arch, wide blade-shaped, outer margin with numerous decumbent spines; loa membranous, elongate finger-shaped, longer than pafa; L4B curved spoon-shaped. Ventral phallomere irregular rhomboidal, posterior margin prominently expanded; sdpl (Fig. 86) more than 90° angle at the middle. **Female** (Figs 72, 74, 77). Similar to male, with following differences. ***Head*** (Fig. 74): width 1.1 to 1.2× as long as head length. Vertex convex. Antenna as long as head to pronotum length. ***Prothorax*** (Figs 76, 77): Pronotum length 3.8–3.9× as long as maximum width; lateral margin with numerous denticles. Prozone dorsal surface with numerous blunted denticles. Metazone 2.8–3.1× as long as prozone. Medial keel protruding, pale brown or occasionally green in green morph. ***Forelegs* (*Prothoracic legs*)**: Coxa dorsal margin with 7–13 large conical spines, small denticles located between them. ***Wings***: Forewing often reaching end of abdomen; discoidal area semi-transparent. **Ootheca** (Fig. 87). ***Measurements* (mm)**: Length 21.8–39.7; maximum width 10.1–13.7; maximum height 7.1–11.0; length of emergence area 15.0–31.0; width of emergence area 3.2–4.3. ***Identification***: Fusiform, nearly hemispherical in cross-section. Proximal end with medial elevation of emergence area. Ootheca attached by ventral surface or fully encircling a thin substrate such as sticks. External wall bright brown. External coating covering almost entire surface of ootheca except lateral zone of emergence area; beige. Lateral side of emergence area prominently concave (Fig. 87). Exhibiting ~ 18–29 egg chambers clearly delimited by visible prominently oblique lips; lips occasionally invisible on new oothecae due to covering by external coating. Distal end of ootheca narrowed to residual process; greatly elongate and attached to substrate. **Nymph. *Mid to last instar nymph***: Avfs 1 and last Avfs base to tibial spur groove with dark spotted pattern; between forecoxa basis membranous attachment surface orange.

##### Biological notes.

*Tenoderaangustipennis* occurs throughout the Korean peninsula and both adults and oothecae can be commonly found on trees and shrubs. First instar nymphs hatch from late May to mid-June, and adults emerge in mid-August.

##### Distribution.

China, India, Japan, Java, South Korea. Invasive in NE USA and Hawaii.

##### Remarks.

*Tenoderaangustipennis* is morphologically similar to *T.sinensis* in Korea but can be distinguished by their more slender bodies, the pronotum length/width ratio, the orange coloration between the forecoxa base (Oshima 2018) (Fig. 81), lack of reddish coloration on the hindwing radius area (Figs 82–84), and the pointed, perpendicular apex of the aafa (Fig. 86).

#### ﻿ Tenodera sinensis

Taxon classificationAnimaliaMantodeaMantidae

Saussure, 1871

5F7AB206-83FF-58C7-B001-3CD354BB51D5

Figs 88–97 , 98–102

Mantis mandarinea Saussure, 1871a: 289.
Tenodera aridifolia var. sinensis Saussure, 1871b: 417.
Tenodera aridifolia (Stoll, 1813): ESK and KSAE 1994: 44 (misidentification). Korean record.
Tenodera sinensis Saussure, 1871: Kim 2010: 31; Kim 2021: 65. Korean record.

##### Specimens examined.

[**KsNU] South Korea: JB**: 1♂, Eunpa Lake, Gunsan-si, 8 XI 2016, Donghwan Na; [**NASIC] South Korea: GW**: 2 Nymphs, Balsan 2-ri, Chuncheon-si, 12 VI 1998, Sungsoon Jang; 1♀, Jinburyeong, Jinbu-myeon, Pyeongchang-gun, 29 IX 2000, Taehwa Kang; 1 Nymph, Ingye-ri, Okgye-myeon, Gangneung-si, 15 VIII 2002, Jingoo Yeo; 3♂, Mt. Odae, Hongcheong-gun, 29 VIII 2019, Jaeil Shim; **GG**: 1♂, Mijang-ri, Samjuk-myeon, Anseong-si, 17 IX 2000, Yeongbo Lee; 1♂, Mt. Cheolma, Incheon, 26 VIII 2001, Taewoo Kim; 1 Nymph, Shihwa, Siheung-si, 30 VII 2003, Jaecheon Son; 1 Nymph, Temple Jeondeungsa, Onsu-ri, Gilsang-myeon, Ganghwa-gun, Incheon, 4 IX 2009, Yeongbo Lee; 1♀, Jikdong-ri, Sohol-eup, Pocheon-si, 7 IX 2011, Yeongbo Lee; 2 Nymphs, Temple Bogwangsa, Gwangtan-myeon, Paju-si, 10 VII 2013, Yeongbo Lee; 3 Nymphs, World Cup Park, Seongsan-dong, Mapo-gu, Seoul, 21 VII 2013, Yeongbo Lee; 2 Nymphs, Noel Park, Sangam-dong, Mapo-gu, Seoul, 30 VII 2013, Yeongbo Lee;); 1♂, Island Gureopdo, Gureop-ri, Deokjeok-myeon, Incheon, 28 VI 2023, Jaeil Shim, Wonjun Sung (reared from nymph); **CB**: 1 Nymph, Magok-ri, Bongyang-eup, Jecheon-si, 13 VII 2005, Taehwa Kang; 1♀, Gamgok-myeon, Eumseong-gun, 31 VIII 2019, Byeongmin Jeong; 3♂, Geumseok-ri, Geumwang-eup, Eumseong-gun, 14 IX 2019, Seong-Gyu Lee; 1♂, Jeongbang-ri, Annae-myeon, Okcheon-gun, 12 X 2000, Yeongbo Lee; **CN**: 1♀, Mt. Sikjang, Daejeon, 14 VIII 2019, Geonheyok Kim (reared from nymph); 4♂, Coastal Dune, Sindu-ri, Wonbuk-myeon, Taean-gun, 12 VIII 2023, Jaeil Shim, near the grassland; **GB**: 1 Nymph, Geumgok-ri, Byeonggok- myeon, Yeongdeok-gun, 1 VII 2009, Yeongbo Lee, Hoyeon Jeong; **GN**: 4♀, Mt. Noja, Dongbu- myeon, Island Geojedo, Geoje-si, 15 IX 2021, Jaeil Shim; **JB**: 1♀, Hoyja 3-dong, Wansan-gu, Jeonju-si, 28 VIII 2014, Taeman Han; 1♂, Deokjin Park, Deokjin-gu, Jeonju-si, 6 IX 2018, Junhee Park; 1♀, Jeonju riverside, Jeonju-si, 15 IX 2018, Jaeil Shim; 1♂, Mt. Moak, Gui-myeon, Wanju-gun, 6 X 2018, Hyeon-Ha Yoo; 1♂, Jinan-Maisan Rest area of expressway, Jinan-gun, 22 VIII 2019, Jaeil Shim; 1♀, Jeonju Univ., Jeonju-si, 4 IX 2019, Jaeil Shim; 1♂, Jangsu-eup, Jangsu-gun, 5 IX 2019, Jaeil Shim; 11♂2♀, Mt. Moak, Gui-myeon, Wanju-gun, 7 IX 2019, Jaeil Shim; 3♂, Temple Geumsansa, Gimje-gun, Gimje-si, 5 IX 2020, Jaeil Shim; **JN**: 1♂, Myeongsasimni, Coastal Dune, Island Bigeumdo, Sinan-gun, 22 IX 2001, Haechul Park; 1♀, Island Gogeumdo, Wando-gun, 3 IX 2003, Mikyung Ahn; 1♂1♀, Bukyi-myeon, Jangseong-gun, 20 VIII 2021, Jaeil Shim;); 2♂1♀, Island Yeoseodo, Yeoseo-ri, Cheongsan-myeon, Wando-gun, VI 2023, Jaeil shim (reared from nymph); **JJ**: 1♀, Ihoteawu Beach, Jeju-si, 26 VIII 2014, Taeman Han, windbreak forest; 2 Nymphs, Gwangchigi Beach, Goseong-ri, Seongsan-eup, Seogwipo-si, 16 V 2021, Jaeil Shim.

**Figures 88–97.**
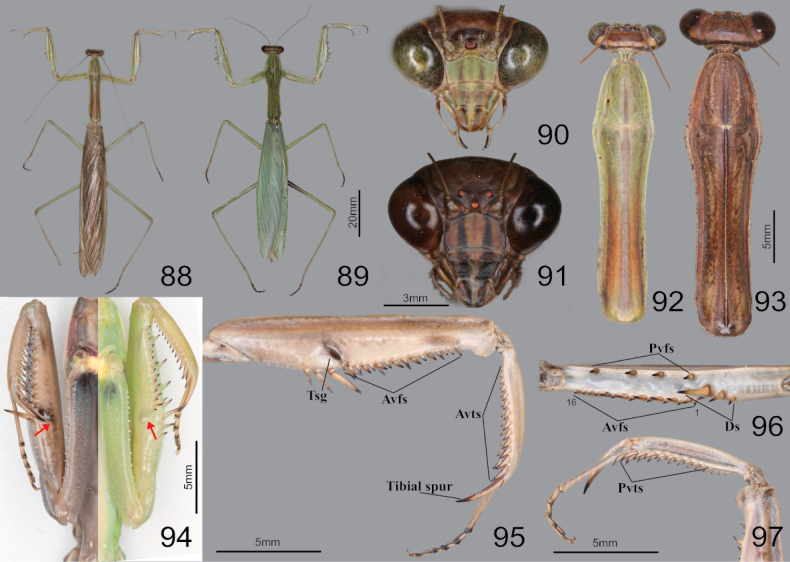
Habitus, head, pronotum and foreleg of *Tenoderasinensis***88** male dorsal aspect **89** female dorsal aspect **90** male face **91** female face **92** male pronotum **93** female pronotum **94** ventral aspect of foreleg and furcasternite (live specimens) **95** foreleg ventral aspect **96** foreleg interior aspect **97** foreleg tibia and tarsus (dorsal aspect). Abbreviation: Tsg = tibial spur groove. Red arrows = dark spot near the tibial spur groove.

##### Redescription.

**Figures 98–102.**
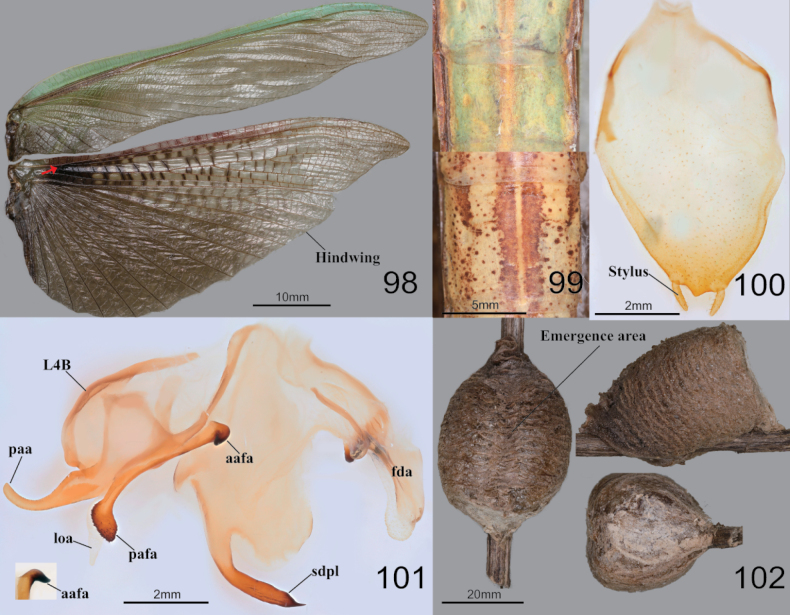
Wings, abdomen, male genitalia and ootheca of *Tenoderasinensis***98** wings **99** male abdomenal sternites (above: green morpho-type below: brown morpho-type) **100** subgenital plate **101** male genitalia (small box = lateral aspect of aafa) **102** ootheca (left: dorsal aspect right above: lateral aspect right below: distal aspect). Red arrow = arculus area.

***Measurements* (mm)**: Total length (vertex to tip of abdomen) ♂ 54.2–89.1, ♀ 58.2–100.8; head width ♂ 5.9–6.5, ♀ 6.9–7.8; head length ♂ 4.5–4.9, ♀ 5.6–6.7; pronotum width ♂ 4.3–5.6, ♀ 4.9–7.4; pronotum length ♂ 18.0–24.3, ♀ 19.2–27.0; forewing (tegmina) length ♂ 38.4–55.2, ♀ 38.1–63.2. **Male** (Figs 88, 90, 92, 95–101). Large to very large. ***Coloration*** (Figs 88, 98): Body and forewing discoidal area green to greenish brown or brown. ***Head*** (Fig. 90): Triangular. Head width 1.2× as long as head. Vertex slightly convex, brownish; apex with a bright brown transverse line (in live specimens). Ocelli large, oblong. Antenna nearly 1.5× as long as pronotum. Epistomal sulcus slightly concave. Lower frons, clypeus, and labrum with two darkish longitudinal stripes. ***Prothorax*** (Fig. 92): Pronotum long but robust, flattened dorso-ventrally, dorsal surface smooth; pronotum length 3.8–4.0× as long as maximum width. Prozone lateral margin and dorsal surface with minute denticles. Metazone (Fig. 92) often orangish; lateral margin smooth; 3.0–3.2× as long as prozone. Between forecoxa basis membranous attachment surface yellow (Fig. 94). Postcervical plate reddish in brown morph (Fig. 94), anterior area of furcasternite with gradational dark pattern in brown morph, its pattern occasionally occurring in green morph. ***Forelegs* (*Prothoracic legs*)** (Figs 94–97): Coxa dorsal margin with 14–17 whitish conical spines; ventral surface of coxa proximal area with gradational dark pattern (Fig. 94); remaining surface with numerous small white spots. Tibial spur groove with faint dark spot (Fig. 94). Spination formula (Figs 95–97): Avts = 14–15; Pvts = 8–10; Avfs = 14–17; Pvfs = 4; Ds = 4. In 16 Avfs (Figs 95, 96): spines 2, 4, 6, 8, 10, 12, and 16 larger than remaining Avfs. Tarsomere distal end dark brown. Tarsi 5-segmented. ***Wings*** (Fig. 98): Forewing completely surpassing the end of abdomen. Forewing costal area green, discoidal area transparent. Hindwing venation brown, cross veins and cells of subcostal to radius area reddish to magenta; radius to cubitus proximal area, near cells of arculus veins dark brown (Fig. 98); anal area with dark brownish smoky pattern. ***Abdomen*** (Figs 99, 100): Middle of the abdominal sternites with longitudinal yellow stripe pattern (Fig. 99). Cerci setose, not flattened, with 17–20 segments. Male subgenital plate (coxosternite IX) (Fig. 100) irregular rhomboidal, inter-stylar margin convex at the middle; ventral surface with numerous setae. Styli rather short. ***Male genitalia*** (Fig. 101): Right phallomere forming nearly V-shaped pva; pia sclerotized and weakly wrinkled; posterior surface of pia (Fig. 101) with membranous wide hump, surface with minute denticulation; fda elongate lobe shape. Left phallomere (Fig. 101) with elongated and curved paa, its apex round; aafa sclerotized, surface smooth, weakly bulbous spike-shaped basally, apically curved dorso-laterally; pafa sclerotized, nearly 90° angle, arched wide blade-shaped (apex expanded), posterior and apical margin of pafa with numerous spines; loa membranous, elongate finger-shaped, much longer than pafa; L4B curved spoon-shaped. Ventral phallomere irregular rhomboidal; posterior margin prominently expanded; sdpl hardly sclerotized, curved at ~ 45° at middle, its distal half slightly thicker and more sclerotized and melanized than basal, point of sdpl tips shallowly concave. **Female** (Figs 89, 91, 93, 94). Similar to male, with following differences. ***Head*** (Fig. 91): width 1.1× as long as head length. Vertex convex. Antenna as long as pronotum. Ocelli smaller than male. ***Prothorax*** (Fig. 93): Pronotum length 2.8–2.9× as long as maximum width; lateral margin with numerous denticles. Prozone with numerous blunt denticles on dorsal surface. Medial keel protruded. ***Forelegs* (*Prothoracic legs*)**: Coxa dorsal margin (Fig. 94) with 14–20 large conical spines (with sharp tips), small denticles located between them. ***Wings***: Forewing occasionally reaching end of abdomen. ***Abdomen***: Elongate oval, much broader than in male. Cerci with 15–17 segments. **Ootheca** (Fig. 102). ***Measurements* (mm)**: Length 30.0–42.2; maximum width 22.2–27.6; maximum height 20.7–24.9; length of emergence area 18.4–32.8; width of emergence area 5.2–5.5. ***Identification***: Barrel-like shape, mostly circular in cross-section. Ootheca attached to flat substrate by its ventral surface or fully encircling substrates such as sticks. External wall with very thick air-filled area, colored beige to bright brown. External coating covering entire surface of ootheca; beige in color. Emergence area depressed. Exhibiting ~ 15–29 egg chambers; lips invisible due to very thick air-filled layer of ootheca. Distal end of ootheca obliquely truncate. **Nymph. *Mid to last instar nymph***: Avfs 1 base (near femoral brush), last Avfs base to tibial spur groove area with dark spotted pattern; between forecoxa basis attachment membranous surface yellow.

##### Biological notes.

*Tenoderasinensis* occurs throughout the Korean peninsula and has adapted well to urban, suburban, and riverside environments. It spawns ootheca in various locations, such as on stones, tree trunks, and branches. First instar nymphs hatch from mid-April to mid-May, while adult mantises typically emerge in mid-August.

##### Distribution.

China, Nepal, Japan, Russia, Thailand, South Korea. Invasive in Canada and the USA.

##### Remarks.

*Tenoderasinensis* was originally described as a variation of the widely distributed species *Tenoderaaridifolia* (Stoll, 1813), and treated as a subspecies for quite a long time (Saussure 1871b; Rehn 1903; Shiraki 1932; Beier 1932; Tinkham 1937; Bazyluk 1977; Iwasaki 1996; Bruins 1999), but already Giglio-Tos (1927) and Ehrmann (2002) considered *T.sinensis* as a valid species. *Tenoderaaridifolia* and *T.sinensis* are extremely close and morphologically very similar (Ehrmann and Borer 2015), but Jensen et al. (2010) provide differences in male genitalia of *T.sinensis* and *T.aridifolia*. According to Jensen et al. (2010), it is a distinctly divided sister species pair based on phylogeny using nuclear genes (histone III, wingless gene) and mitochondrial genes (large and small rRNA, cytochrom oxidase II). *Tenoderaaridifolia* is found in tropical and subtropical regions but is replaced by *T.sinensis* in temperate habitats. Our study used one male West Javan *T.aridifolia* specimen to comparatively examine and recovered remarkable characters from *T.sinensis* including the narrow pronotum (length/maximum width = 5.1) and forewing, a smaller head proportional to body size, short and stout aafa, pafa, and sdpl curved at ~ 45° (Mukherjee et al. 1995; Jensen et al. 2010).

**Subfamily Hierodulinae Brunner de Wattenwyl, 1893**

#### ﻿Genus Hierodula

Taxon classificationAnimaliaMantodeaMantidae

Burmeister, 1838

E3402D15-4FB5-5848-AEC7-4892B6F11241

Parhierodula Giglio-Tos, 1912: 108.

##### Type species.

*Hierodulamembranacea* Burmeister, 1838

##### Diagnosis.

Pronotum clavate. Furcasternite in Korean specimens with spotted pattern or reddish coloration. Forewing stigma triangular, whitish to yellow. Hindwing transparent (Fig. 115). Male subgenital plate margin with numerous black spines. Male genitalia: afa hook- or boat-shaped; maa well developed.

#### ﻿ Hierodula chinensis

Taxon classificationAnimaliaMantodeaMantidae

Werner, 1929

89FA2C3A-8EFA-5D08-BBAD-E986D911D8B9

Figs 103–114 , 115–120

Hierodula chinensis Werner, 1929: 75.
Hierodula chinensis Werner, 1929: Shim et al. 2021a: 121. Korean record.

##### Specimens examined.

[**NASIC] South Korea: CB**: 1 Nymph, Sannam-dong, Seowon-gu, Cheongju-si, 18 VII 2023, NASIC; **GB**: 1♀, Namtong-dong, Gumi-si 10 X 2023, Jaeil Shim; 1♀, Mangudang park, Hyomok-dong, Daegu-si 29 X 2023, Jaeil Shim; **GN**: 2♀, Yonggang-ri, Hwagae-myeon, Hadong-gun, 15 IX 2023, Jaeil Shim; **JB**: 3♂1♀, Jeonbuk Art Museum, Gui-myeon, Wanju-gun, 9 IX 2018, Jaeil Shim; 1♂, Mt. Moak, Gui-myeon, Wanju-gun, 13 IX 2018, Jaeil Shim; 5♂3♀ Mt. Moak, Gui-myeon, Wanju-gun, 7 IX 2019, Jaeil Shim; 6♀, Jeonbuk Art Museum, Gui-myeon, Wanju-gun, 5 IX 2020, Jaeil Shim; 3♂, Jeonbuk Art Museum, Gui-myeon, Wanju-gun, 19 IX 2020, Jaeil Shim; 5♂2♀, National Institute of Agricultural Sciences, Iseo-myeon, Wanju-gun, 20 IX 2023, Jaeil Shim; **JN**: 2 Nymphs 1♂, Cheongso-ri, Seo-myeon, Suncheon-si, 17 VIII 2019, Jaeil Shim; 2 Nymphs, Haesan-dong, Yeosu-si, 20 VII 2022, Jaeil Shim.

**Figures 103–114.**
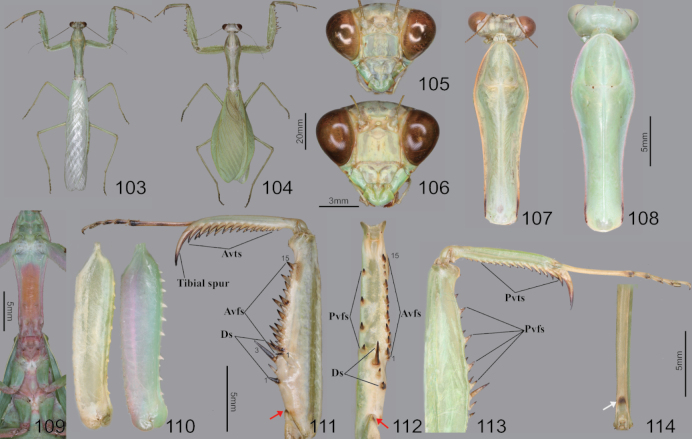
Habitus, head, pronotum, foreleg and hindleg of *Hierodulachinensis***103** male dorsal aspect **104** female dorsal aspect **105** male face **106** female face **107** male pronotum **108** female pronotum (live specimens) **109** furcasternite (live specimens) **110** foreleg coxa ventral aspect (right: male left: live female) **111** foreleg ventral aspect **112** foreleg interior aspect **113** foreleg dorsal aspect **114** hindleg femur (interior aspect). Red arrows = dark spot of foreleg trochanter. White arrow = dark spot of joint.

**Figures 115–120.**
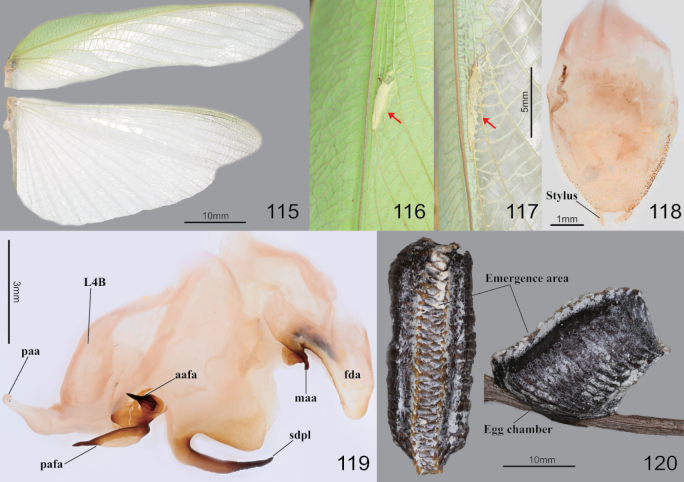
Wings, male genitalia and ootheca of *Hierodulachinensis***115** wings of male **116** female forewing stigma **117** male forewing stigma **118** male subgenital plate **119** male genitalia **120** ootheca (left: dorsal aspect right: lateral aspect). Red arrows = stigma.

##### Description.

See Shim et al. (2021a) for detailed diagnosis and description.

##### Biological notes.

*Hierodulachinensis* is sparsely distributed in the Korean peninsula, primarily inhibiting wooded areas with shrubs and tall trees. It typically lives under tree leaves throughout its lifespan and deposits its oothecae on tree branches. Nymphs have the ability to camouflage themselves by folding their abdomens backwards. The first instar nymphs of *H.chinensis* hatch in early July, with adult mantises typically emerging at the end of August.

##### Distribution.

China. Invasive in Japan and South Korea.

##### Remarks.

*Hierodulachinensis* was recently reported in the Korean peninsula by Shim et al. (2021a). This species was previously recorded in China by Werner (1929) and Beier (1932) and was recently reported in Japan (Yamasaki et al. 2022). However, this species has been erroneously confused with *H.membranacea* Burmeister and *H.macrodentata* Wang, Zhou & Zhang, 2020 by some authors, including Tinkham (1937), Wang (1993), and Zhu et al. (2012). Recent studies by Wang et al. (2020) and Liu et al. (2020) have provided a redescription of *H.chinensis*, clarifying its taxonomic status. See Shim et al. (2021a) for detailed description.

#### ﻿ Hierodula patellifera

Taxon classificationAnimaliaMantodeaMantidae

(Audinet-Serville, 1838)

F6B3A483-C8E6-5836-BC0C-50D67CAECB03

Figs 121–132 , 133–136

Mantis patellifera Audinet-Serville, 1838: 185.
Mantis bipapilla Audinet-Serville, 1838: 188.
Hierodula assamensis Mukherjee et al., 1995: 185.
Hierodula manillensis Saussure, 1870: 233.
Hierodula raptoria Stål, 1877: 38.
Hierodula dispar Kirby, 1900: 146.
Hierodula saussurei Kirby, 1904: 245.
Hierodula manillana Giglio-Tos, 1912: 96.
Hierodula (Hierodula) manillana Giglio-Tos, 1927: 448.
Hierodula yunnanensis Wang, 1993: 137.
Hierodula xishaensis Wang, 1993: 140.
Hierodula multispina Wang, 1993: 141.
Hierodula daqinshanensis Wang, 1993: 143.
Hierodula patellifera (Audinet-Serville, 1838): Jeon et al. 1999: 226 (South Korea); Kim 2010: 31; Kim 2021: 65; Shim et al. 2021b: 149. Korean record.

##### Specimens examined.

[**NASIC] South Korea: GG**: 1♀, Seodun-dong, Suwon-si, 2 IX 1999, Taewoo Kim; 1♂, Seodun-dong, Suwon-si, 13 IV–17 VIII 2001, Taewoo Kim (reared); 4♂1♀, SETEC, Daechi-dong, Gangnam-gu, Seoul, 8 VI 2023, Jaeil Shim (reared from nymph); **CB**: 1♂5♀, Parking area of Cheongju Airport, Ipsang-ri, Naesu-eup, Cheongwon-gu, Cheongju-si, 15 IX 2023, Jaeil Shim; **CN**: 1♂, Rest area, Geumsan-gun, 10 IX 2013, Haechul Park; 1♂, Mt. Gubong, Gwanjeo-dong, Seo-gu, Daejeon, 1 IX 2014, Taekyu Kim; 1♀, Chungnam Nat. Univ., Yuseong-gu, Daejeon, 27 IX 2015, Taeman Han; 4♂2♀, Samsong-ri, Haemi-myeon, Seosan-si, 24 IV 2023, Jaeil Shim (reared from oothecae); 4♂9♀, Chungnam Nat. Univ., Yuseong-gu, Daejeon-si, 19 VIII 2023, Jaeil Shim; **GB**: 2♀, Street near Gomo station (Gomo-ro), Suseong-gu, Daegu, 14 IX 2019, Jaeil Shim; 8♀, Hyomok Elementary Scholl, Hyomok-dong, Daegu-si 29 X 2023, Jaeil Shim; **GN**: 1♀, Mt. Mang, Island Geojedo, Geoje-si, 23 VII 2019, Jun-Gi Lee, Jun-Ho Lee; **JB**: 2♂2♀, Jeonbuk Nat. Univ., Jeonju-si, 21 VIII 2017, Jaeil Shim; 3♀, National Institute of Agricultural Sciences, Iseo-myeon, Wanju-gun, 4 VII 2018, Jaeil Shim (reared from nymph); 2♂, Jangsu-eup, Jangsu-gun, 5 IX 2019, Jaeil Shim; 2♂3♀, Mt. Moak, Gui-myeon, Wanju-gun, 7 IX 2019, Jaeil Shim; 2♂, Jeonbuk Art Museum, Gui-myeon, Wanju-gun, 21 IX 2019, Jaeil Shim; 4♂6♀, Jeonbuk Art Museum, Gui-myeon, Wanju-gun, 22 IX 2019, Jaeil Shim; 2♂10♀, Iseo-myeon, Wanju-gun, 10 IX 2021, Jaeil Shim; : 5♂3♀, Eunpa Lake, Gunsan-si, VI 2022, Jaeil Shim, JuHyeong Sohn (reared from nymph); 2♀, Byeonsan-myeon, Buan-gun, VIII 2022, Jeonbuk Jaeil Shim; 1♂1♀, Hyangga-ro, Pungsan-myeon, Sunchang-gun, 26 VII 2023, Jaeil Shim (reared from nymph); **JN**: 1♂1♀, Near the Korea Coast Guard Academy (KCGA), Yeosu-si, 1 IX 2019, Byeongmin Jeong; 1♂1♀, Dal-dong, Mokpo-si, VIII 2020, Jaeil Shim; **JJ**: 1♀, Jeju Airport, Jeju-si, Jeju-do, 22 IX 2023, Jaeil Shim; 1♂3♀, Donnaeko, Seogwipo-si, Jeju-do, 22 IX 2023, Jaeil Shim; **Guam**: 1♀, Guam, USA, 19 VII 2017, Yeong-Hun Kim; **Japan**: 1♂1♀, Yanabaru, Okinawa, Japan, 1–4 I 2020, Wonjun Sung; **Java**: 1♂1♀, Mt. Argopuro, East Java, Indonesia, V 2019, Jaeil Shim (purchase) **Vietnam**: 1♂, Quang Trung, Bao Loc, Lam Dong, 20 II 2012, Lam Dong Agro-Foresty Research and Experiment Center.

**Figures 121–132.**
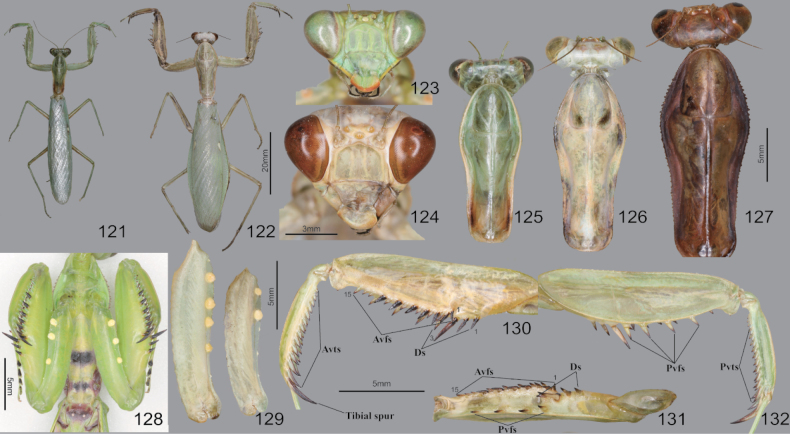
Habitus, head, pronotum and foreleg of *Hierodulapatellifera***121** male dorsal aspect **122** female dorsal aspect **123** male face **124** female face **125** male pronotum (small sized) **126** male pronotum (large sized) **127** female pronotum **128** ventral aspect of foreleg and furcasternite (live specimens) **129** foreleg coxa ventral aspect (right: female with 4 forecoxal spines left: male with 2 forecoxal spines) **130** foreleg ventral aspect **131** foreleg interior aspect **132** foreleg dorsal aspect.

##### Redescription.

**Figures 133–136.**
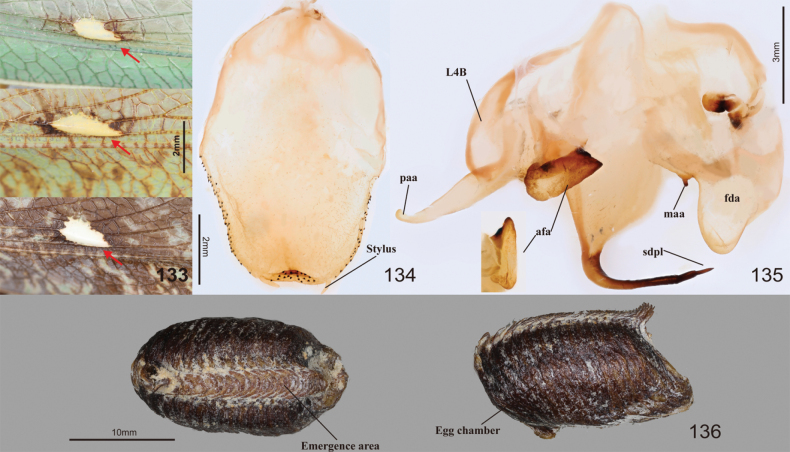
Wings, male genitalia and ootheca of *Hierodulapatellifera***133** stigma of forewing (3 variations) **134** male subgenital plate **135** male genitalia (small box = variation of afa) **136** ootheca (left: dorsal aspect right: lateral aspect). Red arrows = stigma.

***Measurements* (mm)**: Total length (vertex to tip of abdomen) ♂ 44.3–57.8, ♀ 54.3–74.2; head width ♂ 6.6–7.5, ♀ 8.4–9.7; head length ♂ 5.1–5.7, ♀ 7.3–8.1; pronotum width ♂ 5.1–6.0, ♀ 6.7–8.6; pronotum length ♂ 12.9–15.1, ♀ 16.1–20.2; forewing (tegmina) length ♂ 33.8–40.9, ♀ 36.8–47.5. **Male** (Figs 121, 123, 125–126, 129, 133–135). Medium to large sized, body robust. ***Coloration*** (Fig. 121): Bright green to green or bright brown to darkish brown. ***Head*** (Fig. 123): Triangular. Head width 1.3× as long as length. Vertex flat. Compound eye globular, inverse drop-shaped. Ocelli large, oblong. Antenna filiform; length nearly 1.2× as long as pronotum. Lower frons with two very weakly protruding parallel vertical ridges, lower frons width 1.5× as long as height. Epistomal sulcus transverse. Lateral margin of compound eye, mandible, and gena yellow. In live specimens, labrum posterior margin orangish. ***Prothorax*** (Figs 125, 126): Pronotum short, clavate, flatted dorso-ventrally; pronotum dorsal surface smooth and covered in waxy secretion; pronotum length 2.5× maximum width. Prozone lateral margin with numerous denticles. Metazone lateral margin weakly expanded. Medial keel very weakly protruding. Furcasternite (Fig. 128) with two thick transverse and purple markings, larger one at furcasternite medial area and smaller one at posterior one-fourth of furcasternite. ***Forelegs* (*Prothoracic legs*)** (Figs 128–132): Coxa dorsal margin (Figs 128, 129) with 2–5 large spines, rounded triangular or round tooth-like shape, yellow; occasionally 1–3 very minute, white, blunt spines located between large spines. Dorsal and ventral coxal lobes continuous, lacking space between them. Spination formula (Figs 130–132): Avts = 13–15; Pvts = 10–12; Avfs = 14–16; Pvfs = 4; Ds = 4. In 15 Avfs (Figs 130, 131): spines 2, 4, 6, 8, 10, 12 and 15 size larger than remaining Avfs; spines 1, 2, 4, 6, 8, 10 and 12 black. Ds (Fig. 130) 1–3 interior surface black. Tarsomere distal end black. ***Meso- and metathorax and their legs***: Mesothorax sternite anterior area with purple pattern. Meso- and metathoracic legs simple, long, and slender. Tarsi 5-segmented. First tarsomere of midleg slightly shorter than remaining segments combined, first tarsomere of hindleg slightly longer than remaining segments combined. ***Wings*** (Figs 121, 133): Forewing completely surpassing end of abdomen; costal area thick, discoidal area transparent; discoidal area occasionally with brightly colored (yellow, beige, brown) mottled pattern. Stigma (Fig. 133) triangular, white to whitish yellow; rimmed with dark pattern. Hindwing hyaline. ***Abdomen***: Fusiform. Tergites bright yellow to green. Cerci setose, not flattened, with 17 or 18 segments. Male subgenital plate (coxosternite IX) (Fig. 134) irregularly rhomboidal; inter-stylar margin slightly convex and protruding dorsally, margin with 14–20 black spines; ventral surface of subgenital plate with numerous setae; left margin with 41–56 black spines, right margin with 16–28 black spines. Styli rather short. ***Male genitalia*** (Fig. 135): Pia sclerotized and weakly wrinkled; fda triangular; maa short and stout, surface covered by minute spines. Left phallomere (Fig. 135) with elongate and curved paa, its surface smooth, distal area curved dorso-laterally, apex round; afa (aafa+pafa) weakly sclerotized, wide trapezoidal, surface densely covered in minute denticles; anterior margin area of afa, basal one-third with stout dark decumbent projection (aafa); on dorsal surface of afa with long longitudinal groove; posterior apex mostly rounded; loa membranous, weakly humped; L4B curved spoon-shaped. Ventral phallomere (Fig. 135) irregular rhomboidal; sdpl hardly sclerotized, spear and hook-like in shape, curved at basal one-sixth by little more than 90°, remaining distal area long and straight. **Female** (Figs 122, 124, 127–133). Similar to male, with following differences. Body (Fig. 122) robust. ***Head*** (Fig. 124): Vertex slightly convex. Head width 1.1 to 1.2× as long as length. Ocelli smaller than male. Antenna slightly shorter than pronotum. Lower frons width 1.3–1.4× as long as height. ***Prothorax*** (Fig. 127): Pronotum length 2.3–2.4× as long as maximum width; lateral margin with numerous denticles. Medial keel protruding. ***Forelegs* (*Prothoracic legs*)** (Figs 128–132): Large forecoxal spines more pronounced than in male. Number of Avfs = 14–17. ***Wings*** (Figs 122, 133): Forewing occasionally reaching end of abdomen. **Ootheca** (Fig. 136). ***Measurements* (mm)**: Length 21.4–29.0; maximum width 11.2–15.6; maximum height 11.3–13.5; length of emergence area 15.1–21.3; width of emergence area 3.0–3.9. ***Identification***: Oblong, nearly elliptical (mostly dorso-ventrally compressed) in cross-section. Ootheca attached to flat substrate by ventral surface or fully encircling substrate such as sticks. External wall (Fig. 136) dark green to brown. External coating comes off easily in the wild, colored beige. Exhibiting ~ 15–27 egg chambers clearly delimited by visible slightly curved lips. Distal end of ootheca truncate and surface rough.

##### Biological notes.

*Hierodulapatellifera* occurs throughout the Korean peninsula. This species is well-adapted to urban and suburban environments, and can be easily found near mountains, expressway rest areas, and parks. It typically lives under tree leaves throughout its life cycle and lays its oothecae on tree branches, trunks, and building walls near trees. Nymphs fold their abdomens back to camouflage themselves. First instar nymphs hatch in early June and adult mantises emerge in mid-August.

##### Distribution.

China, Guam, India, Japan, Java, New Guinea, Philippines, Sumba, Taiwan, Thailand, Vietnam, Korea. Invasive in France, Italy, and Hawaii.

##### Remarks.

*Hierodulapatellifera* is a widely distributed species (Ehrmann and Borer 2015; Patel and Singh 2016; Shcherbakov and Anisyutkin 2018; Battiston et al. 2019; Moulin 2020). The species exhibits a high degree of morphological variation, which has led to the recognition of numerous synonyms (Audinet-Serville 1838; Wang 1993, Schwarz et al. 2018). Morphological variation, including differences in the number of forecoxal spines, and shapes of forewing stigma and afa, have been observed among *H.patellifera* populations, including those in Korea. Shim et al. (2021b) discuss the challenges of species delimitation in *H.patellifera* in detail and emphasize the need for a comprehensive analysis of both morphological and molecular data to resolve taxonomic uncertainties.

### ﻿DNA barcoding of Korean Mantodea

**Figure 137.**
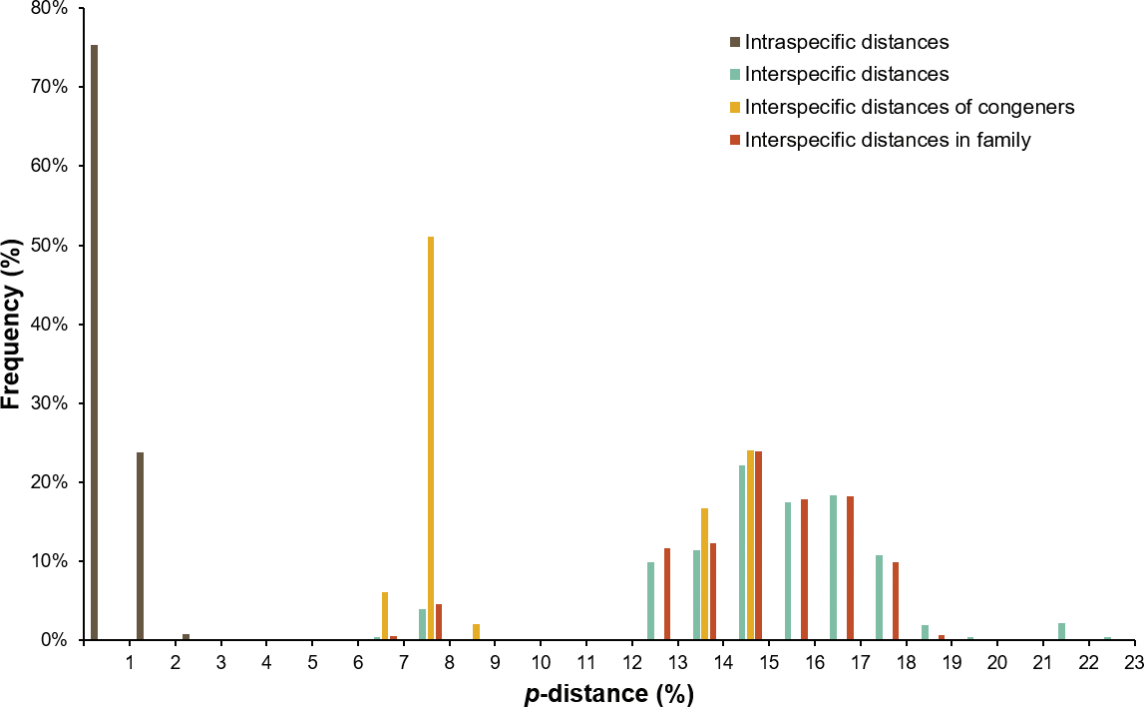
Intra- and interspecific uncorrected distances of partial COI gene sequences for each taxonomic level of Mantodea.

**Figure 138.**
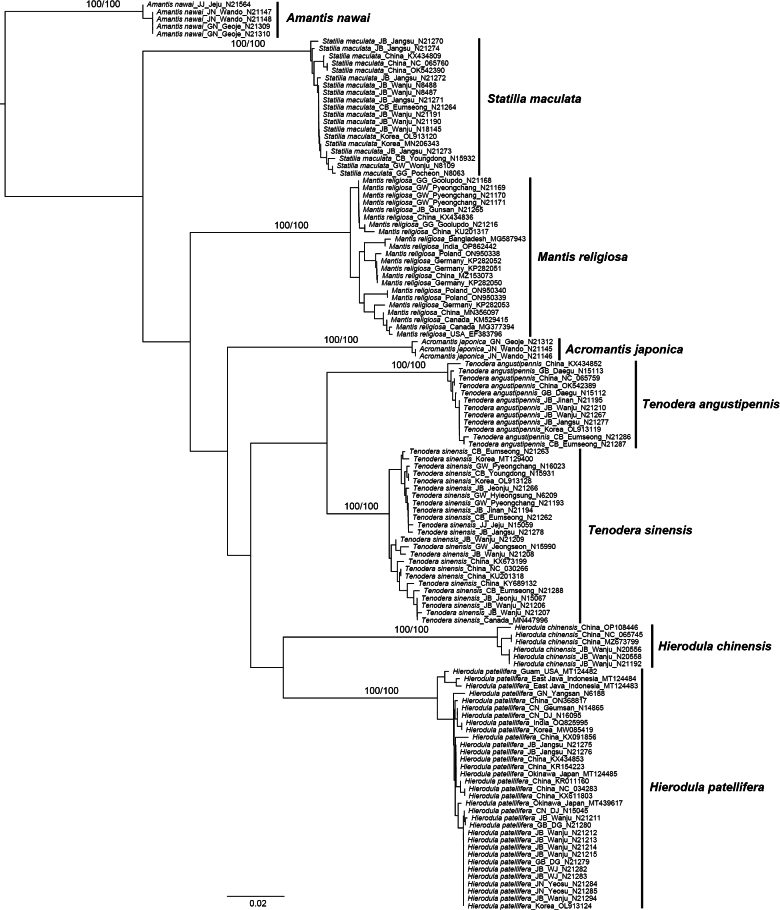
Neighbor-joining tree of Korean Mantodea species based on partial COI gene sequences with bootstrap values (left) and Parsimony analysis bootstrap values (right). Scale bar indicates the expected number of substitutions per site.

In total, 56 new sequences from seven species in six genera were generated (657 bp of COI). All new sequences were deposited in GenBank under the accession numbers OQ826709–OQ826764 (Suppl. material 1). Table 1 and Fig. 137 present the *p*-distances of COI regions for specimens at each taxonomic level. Intraspecific distances from eight species were either identical or very similar (0%–2.2%). The minimum interspecific genetic distance between congeners (6.7%) was ~ 3× higher than the maximum intraspecific genetic distance (2.2%), indicating a significant barcoding gap. All eight species were supported as a single lineage using COI on both NJ and PA trees, respectively (Fig. 138).

**Table 1.** Inter- and intraspecific genetic differences in Korean Mantodea species at each taxonomic level for COI (657 bp) calculated using *p*-distance.

**Taxonomic level**	**Mean**	**SD**	**Max**	**Min**
Intraspecific distances	0.007	0.006	0.022	0.000
Interspecific distances	0.150	0.025	0.224	0.067
Interspecific distances of congeners	0.101	0.033	0.146	0.067
Interspecific distances in family	0.146	0.023	0.188	0.067

## ﻿Discussion

**Figures 139–142.**
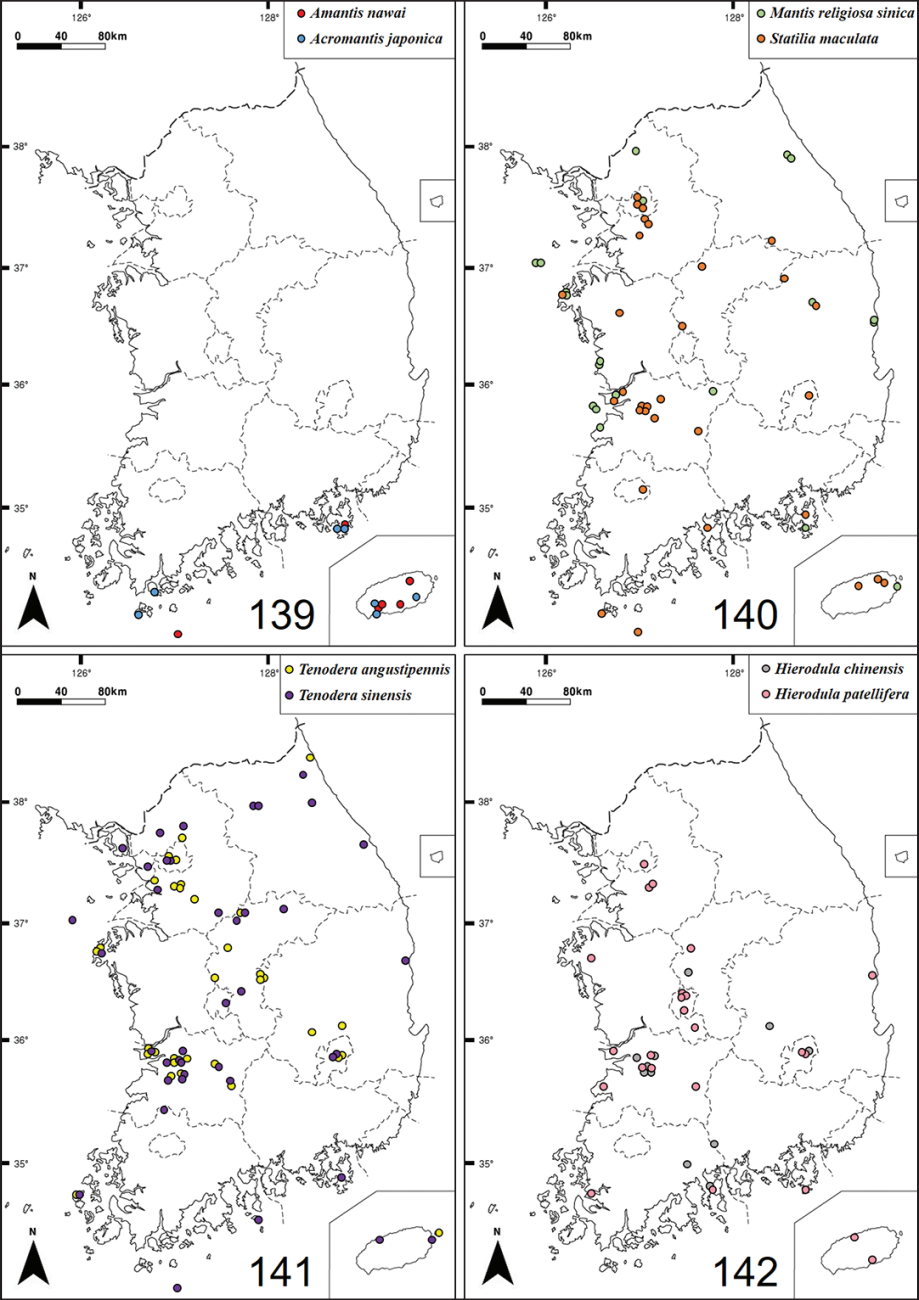
Distribution maps **139***Amantisnawai*, *Acromantisjaponica***140***Mantisreligiosasinica*, *Statiliamaculata***141***Tenoderaangustipennis*, *T.sinensis***142***Hierodulachinensis*, *H.patellifera*.

This study presents the first comprehensive taxonomic review of the Mantodea species in Korea, recognizing eight species based on morphology and DNA barcodes. In contrast to previous studies that primarily focused on the documentation of unrecorded species, our study meticulously examined 494 specimens, encompassing all eight species that have been recorded in Korea. Notably, while the majority of species exhibit a broad distribution across the Korean peninsula (Figs 139–142), the genera *Amantis* and *Acromantis* are confined to the southern islands of Korea (Fig. 139). Furthermore, *Hierodulachinensis*, initially documented in Jeonbuk Province in 2021 (Shim et al. 2021a), has been found in more locations since (Fig. 142).

The morphology of male genitalia is a crucial diagnostic feature for species identification and delineation in mantids, supporting the monophyly of higher taxa (Schwarz and Roy 2019; Liu et al. 2021). However, it is important to note that misidentification at the species level may occur in some cases due to afa structural variations in male genitalia, which can occur intraspecifically in mantids, even under sympatric conditions (Svenson and Roy 2011; Svenson and Vollmer 2014; Shim et al. 2021b). For example, the Korean populations of *Amantisnawai* exhibit two morphotypes of afa structural variations at the intraspecific level, which could lead to confusion at the species level (Figs 16, 17). This situation makes it challenging to determine whether the observed variation in male genitalia represents a cryptic species or a morphological variation. However, the genetic divergence between the two morphotypes based on COI barcode data were not significantly different (0.3%, Table 1), and they were also supported as a single lineage (Fig. 138). This is consistent with our findings that the intraspecific genetic divergence ranges from 0% to 2.2%, while interspecific divergence among congeners ranges from 6.7% to 14.6% (Tables 1, 2; Fig. 137). Furthermore, Mantodea species are each formed as distinct lineages on NJ (Fig. 138) and PA trees (not shown). Consequently, we consider all of them as separate and valid species, given that they exhibit morphological differences as discussed above.

**Table 2.** Inter- and intraspecific genetic differences among Korean Mantodea species for COI (657 bp) calculated using *p*-distance.

	***Amantisnawai***	***Acromantisjaponica***	***Mantisreligiosa***	***Statiliamculata***	***Tenoderaangustipennis***	***T.sinensis***	***Hierodulachinensis***	***H.patellifera***
*Amantisnawai*	0–0.003							
*Acromantisjaponica*	0.165–0.170	0–0.003						
*Mantisreligiosa*	0.159–0.193	0.146–0.178	0–0.022					
*Statiliamculata*	0.155–0.182	0.153–0.160	0.140–0.164	0–0.011				
*Tenoderaangustipennis*	0.179–0.188	0.152–0.161	0.139–0.168	0.168–0.188	0–0.009			
*T.sinensis*	0.165–0.177	0.131–0.143	0.123–0.150	0.142–0.164	0.067–0.083	0–0.017		
*Hierodulachinensis*	0.196–0.204	0.169–0.176	0.146–0.159	0.165–0.182	0.172–0.181	0.143–0.154	0–0.011	
*H.patellifera*	0.210–0.224	0.142–0.153	0.150–0.173	0.163–0.179	0.138–0.154	0.122–0.145	0.134–0.146	0–0.020

Although the diversity of Mantodea in Korea is relatively modest when compared to the high species diversity and endemism observed in neighboring China and Japan (Otte et al. 2024), this study substantially advances our understanding of mantodean diversity within Korea. To further elucidate the diversity of Mantodea across the Korean peninsula, it is imperative that future research initiatives prioritize intensified specimen collection efforts, particularly targeting areas that have been under-sampled to date.

## Supplementary Material

XML Treatment for Amantis

XML Treatment for Amantis nawai

XML Treatment for Acromantis

XML Treatment for Acromantis japonica

XML Treatment for Mantis

XML Treatment for Mantis religiosa sinica

XML Treatment for Statilia

XML Treatment for Statilia maculata

XML Treatment for Tenodera

XML Treatment for Tenodera angustipennis

XML Treatment for Tenodera sinensis

XML Treatment for Hierodula

XML Treatment for Hierodula chinensis

XML Treatment for Hierodula patellifera

## ﻿Additional information

### Author ORCIDs

Jeong-Hun Song https://orcid.org/0000-0003-0317-1457

### Data availability

All of the data that support the findings of this study are available in the main text or Supplementary Information.

## Supplementary materials

Supplementary material 1 List of species with their locality data, voucher numbers, and GenBank accession numbers Data type xlsx Explanation note Asterisks indicate new addition. https://binary.pensoft.net/file/1084009This dataset is made available under the Open Database License (http://opendatacommons.org/licenses/odbl/1.0/). The Open Database License (ODbL) is a license agreement intended to allow users to freely share, modify, and use this Dataset while maintaining this same freedom for others, provided that the original source and author(s) are credited.Jaeil Shim, Jeong-Hun Song
